# Chronological genome and single-cell transcriptome integration characterizes the evolutionary process of adult T cell leukemia-lymphoma

**DOI:** 10.1038/s41467-021-25101-9

**Published:** 2021-08-10

**Authors:** Makoto Yamagishi, Miyuki Kubokawa, Yuta Kuze, Ayako Suzuki, Akari Yokomizo, Seiichiro Kobayashi, Makoto Nakashima, Junya Makiyama, Masako Iwanaga, Takahiro Fukuda, Toshiki Watanabe, Yutaka Suzuki, Kaoru Uchimaru

**Affiliations:** 1 Laboratory of Tumor Cell Biology, Department of Computational Biology and Medical Sciences, Graduate School of Frontier Sciences, The University of Tokyo, Tokyo, Japan; 2 Laboratory of Systems Genomics, Department of Computational Biology and Medical Sciences, Graduate School of Frontier Sciences, The University of Tokyo, Tokyo, Japan; 3 Laboratory of Multi-Omics Data Analysis, Department of Computational Biology and Medical Sciences, Graduate School of Frontier Sciences, The University of Tokyo, Tokyo, Japan; 4 Department of Hematology, Kanto Rosai Hospital, Kanagawa, Japan; 5 Department of Hematology, Sasebo City General Hospital, Nagasaki, Japan; 6 Department of Clinical Epidemiology, Nagasaki University Graduate School of Biomedical Sciences, Nagasaki, Japan; 7 Department of Hematopoietic Stem Cell Transplantation, National Cancer Center Hospital, Tokyo, Japan; 8 Department of Practical Management of Medical Information, Graduate School of Medicine, St. Marianna University, Kanagawa, Japan

**Keywords:** Cancer genetics, Tumour heterogeneity, T-cell lymphoma

## Abstract

Subclonal genetic heterogeneity and their diverse gene expression impose serious problems in understanding the behavior of cancers and contemplating therapeutic strategies. Here we develop and utilize a capture-based sequencing panel, which covers host hotspot genes and the full-length genome of human T-cell leukemia virus type-1 (HTLV-1), to investigate the clonal architecture of adult T-cell leukemia-lymphoma (ATL). For chronologically collected specimens from patients with ATL or pre-onset individuals, we integrate deep DNA sequencing and single-cell RNA sequencing to detect the somatic mutations and virus directly and characterize the transcriptional readouts in respective subclones. Characteristic genomic and transcriptomic patterns are associated with subclonal expansion and switches during the clinical timeline. Multistep mutations in the T-cell receptor (TCR), STAT3, and NOTCH pathways establish clone-specific transcriptomic abnormalities and further accelerate their proliferative potential to develop highly malignant clones, leading to disease onset and progression. Early detection and characterization of newly expanded subclones through the integrative analytical platform will be valuable for the development of an in-depth understanding of this disease.

## Abstract (web summary)

Characterising the clonal architecture of Adult T-cell leukemia-lymphoma (ATL) remains crucial. Here, the authors develop a capture-based sequencing panel and use deep DNA and single cell RNA sequencing and report distinct genomic and transcriptomic features associated with subclonal evolution.

## Introduction

Adult T cell leukemia–lymphoma (ATL) is an aggressive T cell malignancy caused by the human T cell leukemia virus type 1 (HTLV-1). HTLV-1-endemic areas are globally identified, and the number of HTLV-1 carriers has been estimated to be between 5,000,000 and 10,000,000^1^. During the life cycle of HTLV-1, the viral genome is integrated into the human genome, and the expression of viral genes elicits a programmed response from the host genes, which epigenetically leads to the immortalization of infected cells, establishing a polyclonal cell population^2–4^. Thousands of unique infected clones coexist in infected individuals, and many clones persist without transformation to malignant disease. However, after a decade-long asymptomatic period, the human genome accumulates somatic mutations and chromosomal abnormalities^5,6^, which include mutations of critical genes required to induce lymphomagenesis, accompanied by clonal expansion. ATLs are classified into four clinical subtypes: acute, lymphoma, chronic, and smoldering. Chronic and smoldering ATLs are usually indolent types, but most cases eventually progress to aggressive disease. Aggressive ATL is associated with poor prognosis, as indicated by the 4-year overall survival rates of acute-type (11%) and lymphoma-type (16%) ATL^7^. The prognosis of aggressive ATL is generally poor, despite recent advances in treatment modalities^8^. A recent effort employing whole-genome/exome sequencing (WGS/WES) revealed the genetic landscape of ATL^6^. A hallmark of causative driver lesions was their enrichment in the components of the T cell receptor (TCR)/nuclear factor (NF)-κB signaling pathway. Multiple mutations were detected within the signaling pathways involving T cell function and differentiation. Investigations of the clinical effects of genetic abnormalities, based on genotyping data, have supported the significance of genetic profiling for better classification and prognostication in ATL^9–11^. Furthermore, clonally expanded, premalignant cells harboring putative driver mutations were detected in the peripheral blood of individuals at high risk for ATL before clinical onset, indicating that the development of ATL involves the progressive accumulation of mutations within an infected T cell clone^12^. However, the mechanism by which the multiple somatic mutations drive tumorigenesis is not yet completely understood. This is partly due to the lack of research into the relationship between mutations and transcriptome readouts within the ATL cells. It is therefore important to elucidate how and when cell clones propagate to endanger the patient’s life.

Decoding the biological consequences of genomic mutations, first as a transcriptome and eventually as a cellular phenotype, is a central focus of cancer genomics^13^. To address this challenge, the multimodal integration of deep DNA sequencing and single-cell RNA sequencing (scRNA-seq) data is expected to serve as a powerful platform for connecting the causative genomic mutations and resulting transcriptomic outcome at an individual cell level^14–18^. Such a multifaceted use of the scRNA-seq data has been shown to provide valuable insights into the clonal structure of cancer cells. For example, a recent paper on acute myeloid leukemia reported that genomic mutations, such as single-nucleotide variants (SNVs) and indels, could be detected using scRNA-seq reads^16^. Such reads could be utilized to distinguish tumor cells from normal cells and to trace individual cancer cells over time. More importantly, gene expression profiles can be analyzed for mutations in individual cells^16–18^. Similarly, recent efforts connecting other genomic features (e.g., copy number variation) and transcriptome have demonstrated that transcriptional heterogeneity is driven by genetic heterogeneity, resulting in clinical characteristics, such as drug sensitivity and tumor proliferation, in several types of cancer^13,19–21^. Meanwhile, it is still challenging to characterize the transcriptomes of complex mixtures of subclones and precancerous states, because clonal expansion with the genetic alteration is needed. In this regard, other specific molecular signatures, such as cancer-risk associated single-nucleotide polymorphisms (SNPs) and quantitative trait locus (QTL)/expression QTL^22^, or the presence of cancer-associated virus genomes^23,24^ may be useful for the early detection and characterization of cancers.

To understand the heterogeneity and complexity of ATL^11,12^, clone to cell-resolution transcriptome profiling is needed to identify the effects of somatic mutations on the transcriptome during the developmental process of ATL. To evaluate the functional roles of mutations during clonal evolution, longitudinal deep genomic analyses and the influence of genotypes on biological outcomes of transcriptional output are next main challenges. In this study, we developed a sequencing panel designed to facilitate highly sensitive detection and quantification of somatic mutations in ATL cells and alterations in the HTLV-1 genome. Using an integrative genome and single-cell transcriptome platform to connect the clinical timeline, somatic variants^6^, viral transcripts^25^, clonality based on integration sites of provirus^26–28^, tumor-specific surface markers^29,30^, and transcriptome, we characterized clone-specific transcriptomic alterations during intratumoral clonal diversification and mutational evolution over time. In addition, the detection of viral RNA at a single-cell resolution enabled us to characterize the precancerous cell population in asymptomatic individuals.

## Results

### Design of a sequencing panel for genomic mutation analyses of ATL

**Fig. 1:**
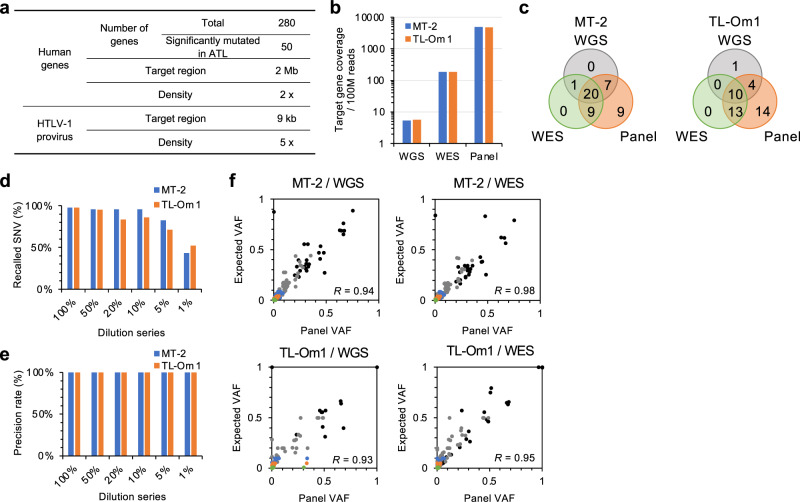
Performance evaluation of HTLV-1/ATL panel for human genome sequencing. **a** Summary of the HTLV-1/ATL panel. **b** Bar graph shows target gene coverage per 100 million reads in different sequencing platforms. **c** Venn diagrams depict overlap between detected SNVs by WGS, WES, and panel-based Target-seq in MT-2 (left) and TL-Om1 (right). **d**, **e** Bar graphs show proportion of recalled SNVs (**d**) and precision rates (**e**) by panel-based deep sequencing from serial diluted samples. SNVs detected by WGS were used as reference. **f** Scatter plots show observed VAF (*x*-axis) and expected VAF (*y*-axis) of detected SNVs in MT-2 (upper) and TL-Om1 (lower). The expected values were calculated from WGS and WES dataset diluted to 100% (black), 50% (dark gray), 20% (gray), 10% (blue), 5% (orange), and 1% (green). Correlation coefficients (*R*) are provided in the graphs.

To design an HTLV-1/ATL panel, we selected 280 human genes, which included 50 genes frequently associated with ATL^6^, and 190 genes frequently mutated in hematological and solid malignancies, available from the COSMIC database^31,32^ and differentially expressed in ATL (GSE33615)^33^ (Supplementary Data 1). The designed panel included 29,068 capture baits for human genes (coding exons + untranslated regions (UTRs), 2× density tiling). To sequence the HTLV-1 genome, we also designed 373 capture baits (5× density tiling), collectively covering the entire provirus. A total of 4849 target regions covering a total length of 2 Mb were targeted (Fig. 1a).

To evaluate the performance of the panel, we selected MT-2 (HTLV-1-infected) and TL-Om1 (ATL-derived) cell lines. The genome of MT-2 harbors multiple proviruses^34^. After enrichment for target regions, genomic DNA was sequenced using the Illumina platform. WGS and WES were conducted to evaluate the performance. SNVs were called according to standard procedures, as described in the “Methods” section. Matched control DNAs were not available for these cell lines; therefore, we removed possible germline SNVs using data from dbSNP and other databases of germline variations.

### Performance of the HTLV-1/ATL panel to detect somatic mutations

We used SNV datasets from different sequencing platforms to compare the coverage and accuracy of mutation calls. Average sequencing depths of ×20, ×300, and ×1700 were obtained from WGS, WES, and panel sequencing, respectively (Supplementary Table 1). Normalized coverage (per 100 M reads) revealed that the HTLV-1/ATL panel afforded ultra-deep sequencing (Fig. 1b). For comparison, we considered SNVs located in the target regions of the panel. Panel-based capture sequencing detected the most somatic mutations called by WGS or WES in both cell lines (Fig. 1c). To determine whether the panel was suitable for a more practical setting, we serially diluted the ATL cell line DNA with normal human DNA. Panel-based ultra-deep sequencing recalled 95.7% (MT-2) and 85.7% (TL-Om1) of SNVs in the presence of 10% abnormal DNA (Fig. 1d). Precision rates determined using SNVs, called using WGS data as reference, were virtually 100% when the ATL genome was ≥1% of input DNA (Fig. 1e). We further evaluated how well the population of abnormal cells represented variant allele frequencies (VAFs). Overall correlations between the expected and observed VAFs were 0.98 (MT-2) and 0.95 (TL-Om1), respectively (Fig. 1f).

### Clonotyping of HTLV-1-infected cell populations using the panel-based deep sequencing

**Fig. 2:**
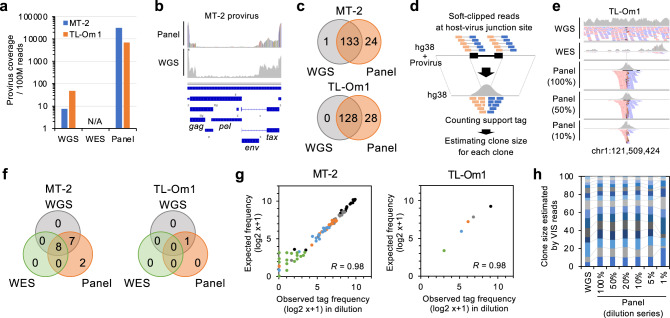
Performance evaluation of HTLV-1/ATL panel for provirus sequencing and clonotyping. **a** Bar graph shows provirus coverage per 100 million reads in different sequencing platforms. N/A not applicable. **b** IGV images depict sequence reads mapped to whole HTLV-1 provirus by panel-based deep sequencing and WGS in MT-2 cells. Internal region with low coverage means defective region. **c** Venn diagrams depict overlap between detected provirus SNVs from WGS and panel-based deep sequencing in MT-2 (upper) and TL-Om1 (lower). **d** A schematic view illustrates clonality assessment by soft-clipped host–virus junction reads. **e** IGV images depict VIS reads called as a dominant clone (chr1:121,509,424) in TL-Om1 by WGS, WES, and panel-based Target-seq (100, 50, and 10% dilution). **f** Venn diagrams depict overlap between detected unique VISs from WGS, WES, and panel-based deep sequencing. **g** Scatter plots show observed clonality (*x*-axis) and expected clonality (*y*-axis). The expected values were calculated from Target-seq (100%) VIS support tags diluted to 100% (black), 50% (dark gray), 20% (gray), 10% (blue), 5% (orange), and 1% (green). Correlation coefficients (*R*) are provided in the graphs. **h** Stacked graph shows comparison of clone sizes calculated by VIS support tags in diluted samples and WGS.

We conducted an evaluation of HTLV-1 genome sequences. Panel-based sequencing yielded >×7000/100 M reads of normalized provirus coverage (Fig. 2a). WES was not applicable for provirus sequencing. Mapped reads, which represented the entirety of the provirus, detected SNVs as well as internal deletions of the provirus, which may not be recognized by the host antiviral immune system^35^. Panel-based ultra-deep sequencing quantitatively detected deletions and mutations with high sensitivity, compared with WGS (Fig. 2b, c). Variants specifically detected by the panel included heterogeneous nonsynonymous and nonsense mutations within the region encoding Tax.

Barcoding with unique virus integration sites (VISs) enables the identification and quantification of thousands of clones, representing virus-associated clonality^26–28^. VISs were detected as reads spanning the human and viral genomes (Fig. 2d; see “Methods” for details). Counts of soft-clipped reads at host–provirus junction sites were used to estimate the population size of each infected clone. We quantified VIS reads and detected multiple VISs at exons, introns, or intergenic regions (Supplementary Table 2). In TL-Om1 cells, one VIS was identified using panel sequencing and WGS (Fig. 2e), consistent with results from a study using fluorescence in situ hybridization and provirus polymerase chain reaction (PCR)^36^. High consistency was achieved when we used VISs detected using WGS as the reference (Fig. 2f).

Similar to the analysis of host mutations, we evaluated the accuracy and sensitivity of detection using sequencing data from serially diluted samples. The overall correlation between the expected and observed clone frequencies was 0.98 for both MT-2 and TL-Om1 (Fig. 2g). The estimated frequency of each clone with a unique VIS was conserved in samples that contained 5% infected cells (Fig. 2h). Thus, the HTLV-1/ATL panel was useful in detecting somatic mutations in both host and proviral genomes and in estimating infected clone size according to the highly sensitive and quantitative identification of VISs. The “viral clonality” was calculated as the population size of each infected clone by counting the reads at the VIS.

### Application of the HTLV-1/ATL panel sequencing for clinical specimens

**Fig. 3:**
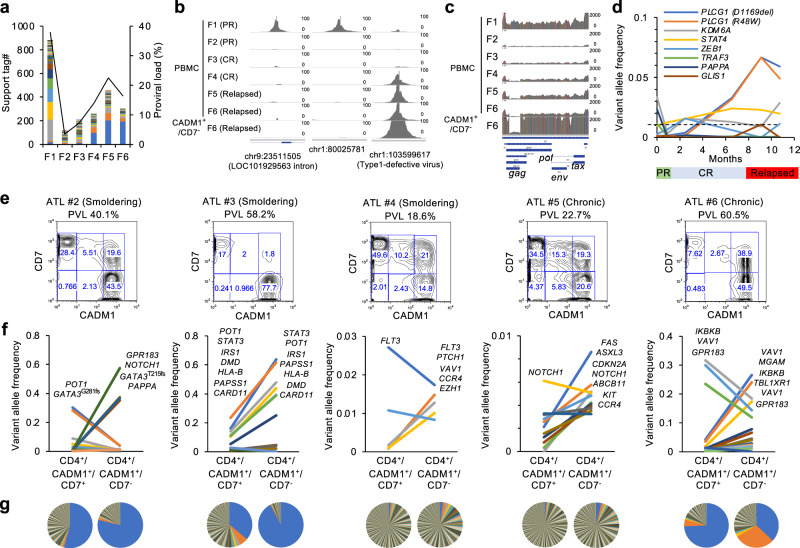
Deep DNA sequencing of clinical specimens from ATL patients. **a** Stacked graph shows chronological transition of clone sizes calculated by VIS support tags in ATL#1. The black line represents HTLV-1 proviral loads (copy per 100 cells). **b** IGV images depict VIS reads called as initial dominant clones (Chr9:23511505, Chr1:80025781) and a relapsed clone (Chr1:103599617) in F1-F6 PBMC and F6 CADM1^+^/CD7^−^ enriched subpopulation. **c** IGV images depict sequence reads mapped to whole HTLV-1 provirus in F1-F6 PBMC and F6 CADM1^+^/CD7^−^ enriched subpopulation. **d** Line plot shows chronological transition of variant allele frequencies of somatic mutations at F1 to F6. **e**–**g** Panels provide results of deep panel sequencing in five indolent ATL cases (ATL#2–6). HAS-flow plots show HTLV-1-infected subpopulation (CD4^+^/CADM1^+^/CD7^+^, “D”), more aggressive subpopulation (CD4^+^/CADM1^+^/CD7^−^, “N”), and HTLV-1-uninfected T cells (CD4^+^/CADM1^−^/CD7^+^, “P”) (**e**). Line charts show VAF values in D and N with annotations of major mutated genes (**f**). Pie charts represent clonalities based on VIS reads in each D (left) and N (right) subpopulations (**g**).

To demonstrate the utility in the clinical setting of the panel developed in this study, we first utilized longitudinally collected genomic DNA from a patient with acute ATL (ATL#1) who received conventional chemotherapy (Supplementary Table 3). Panel sequencing data were collected at the average depth of >×1700. Sequence-tag counts at the virus–host junctions were highly correlated with the estimated frequency of the infected cells (proviral load (PVL); Fig. 3a). The population of each infected clone was estimated by counting the sequence reads spanning the corresponding VISs. This analysis revealed differential compositions of dominant infected clones at disease periods F1 and F4–F6 (Fig. 3b). CD4^+^/CADM1^+^/CD7^−^ ATL subpopulation (see below) at F6 also showed clonal expansion. The clonal expansion of the infected cells was detected as a VIS at chr1:103599617 at F4. At that period, the patient was still clinically complete response, indicating the possibility that the genomic analysis may be able to predict clinical relapse. Mapping to the whole provirus reference genome revealed that the relapsed cells appeared to harbor a defective provirus, which lacked a significant part of the viral genome (Fig. 3c). The sequencing panel also detected multiple somatic mutations. Gain-of-function *PLCG1* nonsynonymous mutations^37^ were detected in advance before clinical relapse (F4), and elevated frequencies were associated with viral clonality and clinical relapse (Fig. 3d).

We further evaluated the performance of panel sequencing using five indolent cases, which may have had a more complex clonal composition (ATL#2–6). We used flow cytometry-associated cell sorting to obtain HTLV-1-infected, phenotypically different subpopulations (CD4^+^/CADM1^+^/CD7^+^ population, designated “D” and CD4^+^/CADM1^+^/CD7^−^ population, designated “N”) (Fig. 3e). According to this HTLV-1 analysis system (HAS-flow), “N” fraction represents more malignant cells^29,30^. Deep sequencing was conducted for each of the subpopulations. Viral clonality and characteristic mutation patterns were identified successfully (Fig. 3f, g). ATL cases #2 and #3 showed a monoclonal expansion. ATL#6 had two expanded infected clones in the CD7-negative N population. Cases harboring expanded infected clones showed somatic mutations with high VAF, such as in *PLCG1* (ATL#1), *GATA3*, *NOTCH1* (ATL#2), *STAT3* (ATL#3), and *VAV1* (ATL#6). In ATL#2, the major VIS detected was identical, but the somatic mutation patterns were mutually different between the D and N fractions, indicating the possibility that the clonal structure switched during disease progression. For ATL#4 and #5, a complicated mixture of multiple variants with low VAF (<0.01) was observed, reflecting the polyclonal nature of the infected cell populations at this stage.

### Transcriptome analyses of ATL specimens

To evaluate the biological relevance of the detected genetic mutations, we analyzed their transcriptome readouts. For this purpose, the genotyped ATL cases (#1, #2, #3, #6) and ATL cell lines (MT-1, *NOTCH1*^P2512L^; ST1 cells, *STAT3*^D566N^; TL-Om1, wild type) were subjected to RNA-seq analyses, first using bulk RNA-seq and then using scRNA-seq. We show how the integrative approach will serve as a powerful approach to reveal the complex cellular and molecular features underlying each of the ATL cases (see below; also see Supplementary Data 2 for the overall summary).

For bulk RNA-seq, a total of 30 M reads per sample was obtained. The RNA-seq detected variant RNAs corresponding to the DNA variant frequencies, including *NOTCH1*^P2514fs^ (gain-of-function mutation^38,39^) in ATL#2_N and *STAT3*^G618R^ (gain-of-function mutation^9,40^) in ATN#3_N (Supplementary Fig. 1a, b). Mutations of the transcription factors (TFs), *NOTCH1* and *STAT3*, are frequently detected in 15.1 and 21.4% of patients with ATL, respectively^6^. However, the way in which these genetic mutations influence their transcriptomes has not been well characterized. The hierarchical clustering of whole transcriptome data showed global changes in the expression patterns (Supplementary Fig. 1c). In addition, genes differentially expressed on a case-specific basis were also detected (Supplementary Fig. 1d, e). To investigate the biological relevance of the mutations on the altered transcriptome, we looked for the target genes of these TFs, NOTCH1 and STAT3, using the chromatin immunoprecipitation sequencing (ChIP-seq) database (described in “Methods”). We found that some of their key target genes were upregulated in the corresponding mutant cells (Supplementary Fig. 1f, g). Representative TF binding and the specific transcription of target genes (NOTCH1 target *HES4*^41^; STAT3 target *SOCS3*^42^) are provided using the Integrative Genome Viewer (IGV; Supplementary Fig. 1h).

Substantial information could be obtained from the integrative analysis of panel genomic sequencing analysis (Fig. 3) and bulk transcriptome analysis (Supplementary Fig. 1). However, further detailed analysis was needed to elucidate the transcriptomic consequences of the genomic mutations in subpopulations. It was also necessary to investigate whether the subclonal structure identified based on genomic profiling was correct. Therefore, we decided to conduct a systematic scRNA-seq analysis.

### Detailed detection of RNA variants and viral transcripts using scRNA-seq

To reveal the transcriptome figures of complex mixed subclones, beyond the averaged bulk analysis, we employed scRNA-seq analysis using the 10× Genomics Chromium platform. For this analysis, we utilized longitudinal specimens at two timepoints, T1 and T2, from three indolent ATL cases (ATL#2, #3, #6; Supplementary Table 3 and Supplementary Data 2). In one case, ATL#6, we generated six scRNA-seq libraries from three subpopulations (P, D, and N) at T1 and T2, respectively, to detect intratumoral heterogeneity and transition. For each scRNA-seq dataset, a total of 22,400 genes was represented by 115,567 sequence reads per cell. The statistics of the scRNA-seq datasets are summarized in Supplementary Data 3. We sought to identify cells expressing any of the transcripts harboring the detected genomic somatic variants. Referring to the previous study^16^, we labeled a cell as “mutant” if it contained at least one variant read, and “wild-type” if only wild-type reads were detected. We evaluated the detection rate of the variants by comparing the bulk and single-cell RNA-seq platforms and detected 88.1% of the expressed RNA variants using this scRNA-seq platform. An average of 14.9 mutant cells per RNA variant was detected. Variants of varying frequencies were represented, including founding clone mutations, subclonal mutations, and putative driver mutations, for a variety of variation types, such as SNVs in *VAV1*^Y174C/M501R^ and *PRKCB*^D427N^, and indels in *GATA3*, *CARD11*, and *HLA-B*. In addition, we successfully compiled viral reads from scRNA-seq data. HTLV-1 antisense RNA (*HBZ*), which is stably expressed in the infected cells, was detected in all cases, with an average detection of 544 cells per sample (11.1% of the estimated total infected cells). Infected clone-specific host–virus chimeric reads^43^ were also detected as scRNA-seq reads spanning the VIS from an average of 19 infected cells (see “Methods”).

### Characterizing subclone-specific transcriptome consequences of genomic mutations: the case of ATL#6

**Fig. 4:**
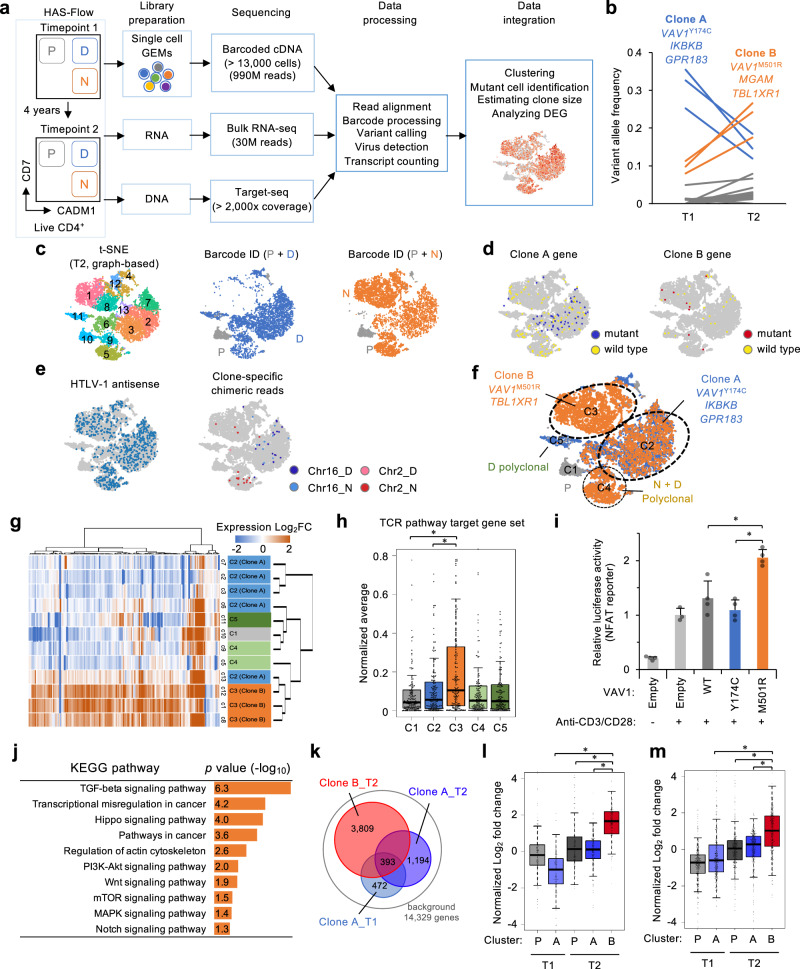
Single-cell transcriptome analysis combined with deep sequencing for characterization of subclonal population. **a** Workflow shows the collection and processing of fresh peripheral blood samples from indolent ATL, followed by scRNA-seq/deep sequencing platform. **b** Line plot shows transition of VAFs at T1 to T2 in CD4^+^/CADM1^+^/CD7^−^ population in ATL#6. Each line represents transition of VAF values of mutated genes during T1 to T2 period. Putative driver genes in Clone A (blue) and Clone B (orange) are annotated. **c** *t*-distributed stochastic neighbor embedding (*t*-SNE) projection from P, D, and N subpopulations (total 13,087 cells) reveals graph-based clusters. The clusters are assigned to the indicated phenotypes by cell barcode ID. **d** *t*-SNE plots with single-cell mutation detection. Cells colored according to single-cell genotype at Clone A genes (*VAV1*^Y174C^, *IKBKB*, and *GPR183*) and Clone B genes (*VAV1*^M501R/V^ and *TBL1XR1*): blue and red, at least one mutant read detected; yellow, wild-type reads only; gray, no coverage. **e** *t*-SNE plots with viral antisense RNA (left) and clone-specific chimeric transcripts at Chr16 and Chr2 (right). **f** *t*-SNE plot shows large clusters (C1 to C5) as identified by differentially expressed marker genes, distribution of cell phenotype, detection of mutated RNA and clone-specific chimeric RNA, and population size estimated by VIS reads. **g** Clustered heatmap depicts expression levels (Log_2_ fold change) of TCR pathway target genes. **h** Box plot shows normalized average of expression levels of TCR pathway target genes (167 genes from GSE13738 dataset) in clustered populations. The middle lines within box plots correspond to the medians; lower and upper hinges correspond to first and third quartiles. The upper whisker extends from the hinge to the largest value no further than 1.5 × IQR. The lower whisker extends from the hinge to the smallest value at most 1.5 × IQR. All data points are overlaid on the box plot. *p ≤ 0.05 (two-sided Student’s *t* test). **i** Bar graph shows reporter-based NFAT activity in Jurkat cells expressing wild-type or mutant VAV1 in the presence of TCR engagement. *n* = 3–4 biologically independent samples, mean ± SD, *p ≤ 0.05 (two-sided Student’s *t* test). Raw data are available from Source data file. **j** Bar graph shows enriched KEGG pathways with one-sided Fisher’s exact *p* values (−Log_10_) in C3 cluster cells compared with C2 (Log_2_FC ≥ 2 in C3, 2234 genes). **k** Venn diagram depicts overlapped and clone-specific genes in Clone A and Clone B at T1 and T2 (FC ≥ 2 vs. P subpopulation, *p* ≤ 0.05). **l**, **m** Box plots show normalized Log_2_ fold changes of TCR pathway target genes (167 genes from GSE13738 dataset) (**l**) and cell-cycle process genes (377 genes) (**m**) in clustered subpopulations at T1 and T2. P, uninfected T cells. The middle lines within box plots correspond to the medians; lower and upper hinges correspond to first and third quartiles. The upper whisker extends from the hinge to the largest value no further than 1.5 × IQR. The lower whisker extends from the hinge to the smallest value at most 1.5 × IQR. All data points are overlaid on the box plot. *p ≤ 1 × 10^−5^ (two-sided Student’s *t* test).

We attempted to demonstrate the way in which integrative genomic and transcriptomic analyses could contribute to understanding clonal expansion and the occasional switch of the dominant clones. For ATL#6, we first analyzed the subclonal structure using panel sequencing (Fig. 4a and Supplementary Fig. 2a–d). The viral clonality indicated a relative expansion of the infected clone with type 2 defective virus integrated into Chr2 during the T1 to T2 period. The PyClone algorithm^44^, based on somatic mutation data, predicted the coexistence of two major clones. Clone A (full-length virus integrated into Chr16) represented the major population with *VAV1*^Y174C^, *IKBKB*, and *GPR183* mutations in the fractions D and N at T1. Clone B (type 2 defective virus integrated into Chr2) harboring *VAV1*^M501R^, *MGAM*, and *TBL1XR1* mutations was detected in the N fraction as a minor population at T1. However, this mutationally defined clone had expanded by T2 (Fig. 4b). Several lines of evidence supported the idea that the subclonal structure transitioned from Clone A at T1 to the emerged Clone B. A bulk TCR repertoire analysis also detected the clonal propagation of a T cell clone having TCR Vβ13.1, which corresponded to Clone B, providing further evidence that the dominant T cell clones changed between T1 and T2 (Supplementary Fig. 2e, f).

Data from 13,087 high-quality single-cell transcriptomes obtained from the subpopulations at T2 were analyzed (Fig. 4c). The distribution of the barcode IDs and the characterization of marker genes identified five large clusters, C1–C5 (Supplementary Fig. 3a–d). In particular, the C3 cluster comprised CADM1^+^/CD7^−^ (N) cells (94.5%). Frequencies of C2 and C3 were consistent with those of Clone A and Clone B, as estimated by VIS reads. To further confirm the correspondence of the clones, we highlighted cells harboring genomic mutations (Fig. 4d and Supplementary Fig. 3e, f). The corresponding mutations of Clone A genes and Clone B genes were detected in C2 and C3, respectively (*p* ≤ 0.05, one-sided Fisher’s exact test). In addition, we overlaid HTLV-1 reads on the clusters (Fig. 4e and Supplementary Fig. 3g, h). We found the stable expression of HTLV-1 antisense RNA (predominantly *HBZ*) specifically in D (6.57% of total cells) and N (7.52%) subfractions, compared to P (0.15%). Furthermore, infected clone-specific host–virus chimeric reads were significantly enriched in these clusters (*p* ≤ 0.05), validating that those cells are really infected cells (Fig. 4f).

Both Clone A and Clone B (or C2 and C3, respectively) showed changes in their transcriptomes bearing the hallmarks of enhanced proliferation. We analyzed the expression of the TCR–NF of activated T cell (NFAT) pathway target genes (GSE13738), as this is the central pathway involved in the control of the proliferation of ATL cells. VAV1, in which genomic mutations were found for both Clone A and Clone B, is involved in this pathway^45^. When we examined subclone-specific transcriptome features, we found that Clone B (C3) with *VAV1*^M501R/V^ exhibited a relative upregulation of the downstream target genes of the TCR pathway compared with Clone A (C2) and other clusters comprising polyclonal infected cells or uninfected T cells (Fig. 4g, h). We further conducted a NFAT reporter assay using in vitro cell culture. We found that the ectopic expression of VAV1^M501R^ exhibited elevated NFAT activity compared with VAV1^WT^ and even to the greater extent than VAV1^Y174C^ in the presence of TCR engagement (Fig. 4i). Furthermore, we analyzed NFAT ChIP-seq data to identify NFAT targets in TCR-engaged CD4^+^ T cells (Supplementary Fig. 4). We found that Clone B expressed higher levels of NFAT target genes, including well-characterized TCR pathway component genes (e.g., ZAP70, *BCL10*, *NFKB2*) and anti-apoptotic genes (e.g., CFLAR, *BIRC2/3*, *MCL1*). Lastly, we found that scRNA-seq data also indicated an activation of other important signaling pathways, including PI3K-AKT and transforming growth factor-β (TGF-β) pathways (Fig. 4j and Supplementary Fig. 5). Gene set enrichment analysis (GSEA) revealed that the enrichment was associated significantly with cell proliferation (Supplementary Fig. 6). Cell-cycle genes, including *E2F2, MKI67, CCNA2*, and *CCNT1*, were upregulated in Clone B (C3). In contrast, Clone A (C2) comprised molecular signatures, such as oxidative phosphorylation, DNA repair, and fatty acid metabolism. Thus, the distribution of clustered cells showed differential characteristics within intratumoral heterogeneity. Collectively, these data indicated that a specific *VAV1* mutation contributed to NFAT activation and led to the development of Clone A and Clone B. It also appeared that Clone B should have more aggressive features than Clone A, which may explain the molecular mechanism of the competition between the subclones.

### Transcriptome evolution over time: the cases of ATL#2, #3, and #6

We characterized the transcriptome with respect to the evolutionary dynamics of clonal expansion. To detect the biological significance of the genetic mutations, we analyzed longitudinal alterations (T1 to T2) of the transcriptomes of major clones in three clinical cases, which harbored key mutations frequently detected in ATL patients^6,10,11^: *VAV1* (ATL#6), *NOTCH1* (ATL#2), and *STAT3*/*PRKCB* (ATL#3). All cases showed the relative expansion of specific clones harboring specific somatic mutations between T1 and T2, an average 2.6-year interval (Supplementary Table 3 and Supplementary Data 2). Two of the three cases showed additional mutations at T2 (ATL#3, 6). In the scRNA-seq analysis, the peripheral blood mononuclear cell (PBMC) samples collected at T1 and T2 were analyzed and then integrated with serial data for direct comparison. Key features, such as timepoints, viral transcripts, host–virus chimeric reads, host mutations, and specific marker genes, showed the presence of major clone clusters for these cases (*p* ≤ 0.05). Thus, we followed the history of individual clones over time.

For ATL#6, 32,813 single-cell data collected at T1 and T2 were analyzed together (Supplementary Fig. 7). T1 data did not show clear intratumoral heterogeneity. However, at T2, we detected a large number of upregulated genes (3809 genes, *p* ≤ 0.05) with clonal advantage over other cells in the emerged Clone B at T2 (Fig. 4k). The altered gene set included multiple genes associated with the TCR pathway and cell proliferation processes, suggesting the functional relevance of *VAV1*^M501R^ in clonal expansion (Fig. 4l, m).

In ATL#2, a cluster of dominant cells with *NOTCH1*/*GATA3*/*GPR183* mutations was identified. The genomic mutation-based inference of clone composition also indicated a continuous expansion of this genetically identical clone (Supplementary Fig. 8a, b). The *NOTCH1* variant may be one of the driver mutations, although this mutation was not directly detected by scRNA-seq, perhaps due to its low expression level. Instead, the other major variants (*GATA3* and *GPR183*), which were predicted to form the same cluster by genomic inference, were detected (Supplementary Fig. 8c–h). In this case, the efficiency of detection of the mutation was not high, but viral transcripts and chimeric transcripts supported the cluster inference (*p* ≤ 0.05). Correlations in the expression levels of some NOTCH1 target genes were detected for this cluster (*p* ≤ 0.05, Supplementary Fig. 8i). In this case, cells at T2 showed no additional mutations and were assigned to the same cluster as cells at T1. Nevertheless, a comparison of the transcriptomes at T1 and T2 identified the upregulation of 1106 genes in cells at T2 (*p* ≤ 0.05). Genes associated with the cell-cycle process and interleukin-1 signaling were enriched, indicating that gene expression changes without explicit genomic mutation changes may be responsible for the progression of the clone over this timeline (Supplementary Fig. 8j). Integration with ChIP-seq peak-call data also suggested that several NOTCH1 target genes were involved in the enhanced cell-cycle process (Supplementary Fig. 8k).

To further validate the activity of the mutated *NOTCH1*, we surveyed ATL-derived cell lines using panel sequencing and RNA-seq and found an analogous mutation in *NOTCH1* in MT-1 cells (Supplementary Fig. 9a). MT-1 cells also contained a stop-gain mutation in *CBLB*, which encodes the E3 ubiquitin ligase of NOTCH1^46,47^. Treatment with γ-secretase inhibitor (GSI) significantly reduced the level of intracellular NOTCH1 (ICN1) and suppressed the transcription of NOTCH1 target genes, some of which were upregulated in ATL#2 scRNA-seq data (Supplementary Fig. 9b, c). We found that cellular proliferation was subsequently inhibited, as expected (Supplementary Fig. 9d).

**Fig. 5:**
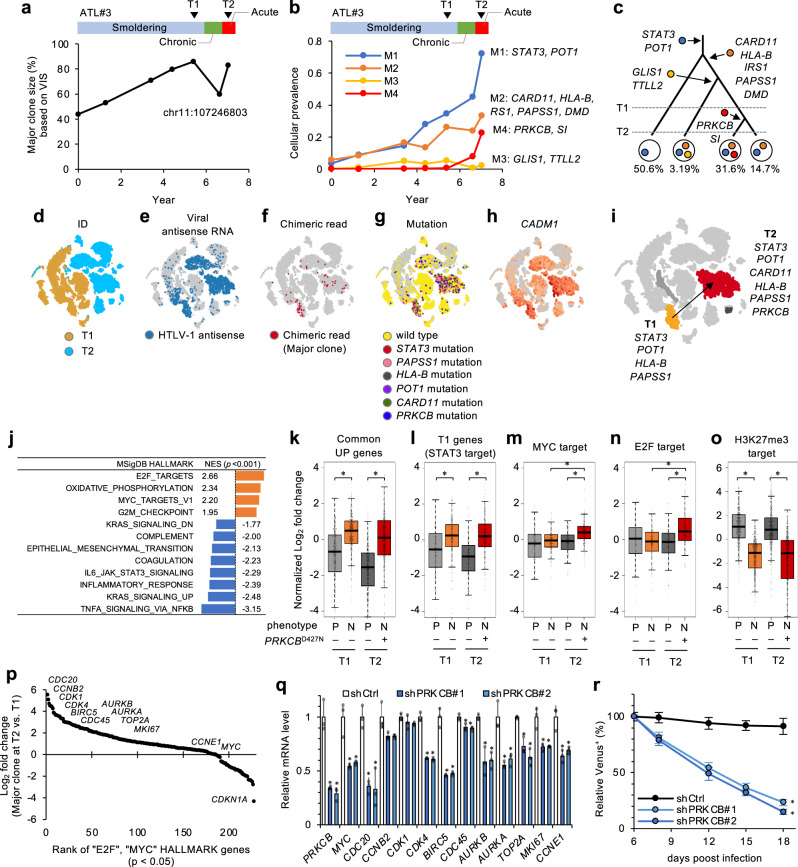
Mutation-associated transcriptomic evolution. **a** Transition of a major clone size calculated by VIS support tags in ATL#3. **b**, **c** Clonal evolution in ATL#3. Line plot shows chronological transition of cellular prevalence calculated from the PyClone model (**b**). Subclonal information with imputed mutational subclusters is depicted in phylogenetic tree (**c**). PBMCs at the indicated timepoints (T1 and T2) were subjected to the longitudinal scRNA-seq analysis. **b**–**i** *t*-SNE projections of scRNA-seq data in ATL#3 with cells colored according to timepoints (**d**) viral antisense RNA (**e**), clone-specific chimeric transcripts (**f**), single-cell mutation detection (**g**), and *CADM1* expression (**h**). The assigned major clone clusters at T1 (orange) and T2 (red) and evolutionary trajectory are illustrated on *t*-SNE (**i**). The mutated genes detected in scRNA-seq are annotated. **j** Table summarizes normalized enrichment scores (NES) of significant hallmarks (one-sided *p* ≤ 0.001) in *PRKCB*^D427N^ subclone at T2. **k**–**o** Box plots show normalized Log_2_ fold changes of commonly upregulated genes (511 genes) and case-specific genes at T1 (265 genes) (**k**, **l**, both defined in Supplementary Fig. 1), MYC_TARGEST_V1 (196 genes) (**m**), E2F_TARGETS (198 genes) (**n**), and H3K27me3 targets (608 genes) (**o**) in the indicated clusters at T1 and T2. The middle lines within box plots correspond to the medians; lower and upper hinges correspond to first and third quartiles. The upper whisker extends from the hinge to the largest value no further than 1.5 × IQR. The lower whisker extends from the hinge to the smallest value at most 1.5 × IQR. All data points are overlaid on the box plot. *p ≤ 1 × 10^−5^ (two-sided Student’s *t* test). **p** Scatter plot shows expression score (Log_2_FC) of HALLMARK E2F and MYC targets (two-sided *p* < 0.05) in *PRKCB*^D427N^ subclone at T2. **q**, **r** Functional analysis of *PRKCB*^D427N^. *PRKCB* expression was depleted by shRNA in *PRKCB*^D427N^-expressing KOB cells. mRNA levels of cell proliferation-associated genes (**q**) and results as percentage of each starting Venus-positive population of KOB cells transduced with shCtrl, shPRKCB#1, or shPRKCB#2 (**r**) are shown. *n* = 3 biologically independent samples, mean ± SD, *p ≤ 0.05 (two-sided Student’s *t* test). Raw data are available from Source data file.

ATL#3, which harbored a *STAT3* mutation at T1 (Fig. 3), showed rapid expansion of a major clone by gaining an additional *PRKCB*^D427N^ mutation during its acute progression (summarized in Supplementary Data 2). This *PRKCB* mutation is known to be a poor prognostic hotspot mutation in aggressive ATL^9^. The transition of viral clonality and Bayesian clustering of somatic mutations proposed a “linear evolutionary model” with branched subclones (Fig. 5a–c). The newly emerged subclone with *PRKCB*^D427N^ may have drastically propagated widely within half-a-year. T1/T2 scRNA-seq data with highlighted features (somatic variants, viral transcripts, host–virus chimeric reads, and expression levels of specific marker genes) also collectively represented the rapid clonal expansion during T1 to T2 (Fig. 5d–i). The scRNA-seq detected variant RNAs corresponding to DNA variant frequencies, which assigned clusters (*p* ≤ 0.05; Supplementary Fig. 10). In addition, 8 cells (0.1%) harbored multiple mutations in key genes (*PRKCB*/*STAT3*, *PAPSS1*/*STAT3*, *STAT3*/*HLA-B*, *PAPSS1*/*HLA-B*) in the scRNA-seq dataset. The observed mutation combinations were consistent with the subclonal structure predicted by the genome data.

GSEA showed the enrichment of cell growth hallmarks (E2F and MYC targets) in this *PRKCB-*mutated clone (Fig. 5j). Transcriptome data from subclusters demonstrated the sustained expression of founder genes identified at T1, including commonly upregulated genes and STAT3 targets, and further acquisition of cell growth hallmarks in the *PRKCB*^D427N^ subclone (Fig. 5k–o), suggesting that transcriptomic characteristics are accumulated in accordance with a process of multistep mutagenesis. Notably, H3K27me3-mediated epigenetic silencing^4,48^ was commonly detected at T1 and T2. Inflammatory genes were significantly suppressed in the evolved *PRKCB*^D427N^ subclone.

Particularly for this clone, we conducted additional biological analyses. *PRKCB*^D427N^ is known to cause the hyper-activation of NF-κB^6^. In fact, rank ordering of hallmark genes showed upregulation of cell-cycle genes in the *PRKCB*^D427N^ mutant clone (Fig. 5p). To validate the function of *PRKCB*^D427N^, we performed additional sequencing and found that ATL-derived cell line KOB had similar *PRKCB*^D427N^ mutation and transcriptome features. In KOB cells, the specific knockdown of *PRKCB* genes resulted in a decreased expression of MYC and other genes associated with cell proliferation and eventually led to lower cell proliferation (Fig. 5q, r).

Collectively, we successfully demonstrated the power of the analytical platform. Through a series of analyses of cases with representative ATL-type mutations, we found that characteristic somatic mutations should have distinct clone-specific transcriptomic features, acquired from additional somatic mutations or the modification of transcriptome networks over time. We detected specific expression changes and differential transcriptomic features in mutationally defined clones among clinical cases with different genetic drivers. On the other hand, genes associated with cell proliferation were commonly upregulated during clonal expansion in all clinical cases.

### Longitudinal genomic profiling of asymptomatic HTLV-1 carriers: the cases of AC#1–10

**Fig. 6:**
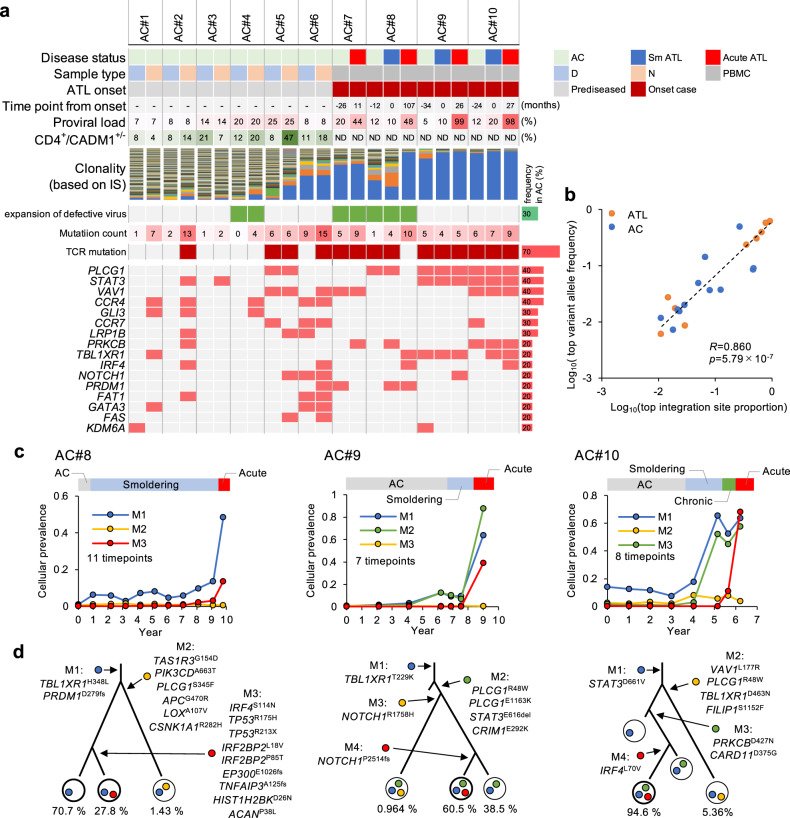
Longitudinal genomic profiling of asymptomatic HTLV-1 carriers. **a** Overview of the clonality and somatic mutations across 10 asymptomatic carriers (ACs). For #1–6, peripheral blood samples from asymptomatic donors were subjected in HAS-flow sorting to collect D and N subpopulations for quantifying very low-frequent mutations from polyclonal populations. For ATL progression cases (#7–10), PBMCs before and after diagnosis were subjected to deep sequencing. TCR pathway mutation was defined by detection of *PLCG1*, *VAV1*, *PRKCB*, or *CARD11* mutation (≥1) in major clones. Clonal mutations are defined as mutations with VAF ≥ 0.01. Detailed detected mutations are provided in Supplementary Data 4. **b** Scatter plot shows major clone sizes estimated by VIS read counts (*x*-axis) or VAF values (*y*-axis) for each sample (Log_10_). A two-sided Spearman test was performed (*n* = 21 samples). **c**, **d** Clonal evolution in disease progression cases (AC#8–10; follow-up period, 6–9 years). Collected samples included PBMCs at AC (before onset), indolent ATL, and after acute transformation. Line plots show chronological transition of cellular prevalence of imputed mutational subclusters (**c**). Subclonal information and imputed mutational subclusters in each sample are depicted in phylogenetic trees (**d**).

A better understanding of ATL, especially in the early stages, is expected to provide the basis for pre-emptive therapeutic strategies for this poor prognostic disease. To this goal, we prospectively analyzed somatic mutations and the clonal structure of virus-infected cells from pre-onset individuals (asymptomatic HTLV-1 carriers) (Fig. 6). Ten HTLV-1 carriers, including four (AC#7–10) with ATL progression over nearly 10 years, were analyzed using genomic panel sequencing. For the disease progression cases, PBMCs collected before and after diagnosis were used. For the asymptomatic donors (AC#1–6), the CADM1^+^ subfraction was available to quantify low-frequency mutations from polyclonal populations. Overall, we expected that useful information could be obtained from such archival samples, even though fresh blood samples, which are needed for scRNA-seq analysis, are not always available.

In the genomic sequence data, VIS reads showed clonal expansion in cases with ATL progression (#7–10) before and after onset, which is consistent with a previous study^12^. In addition, two asymptomatic carriers (ACs) (AC#5, #6) also exhibited clonal expansion, with the top dominant infected clone accounting for ≥10% of infected cells, even though this expansion preceded the clinical appearance of the condition.

Several somatic mutations in key genes were detected in all AC cases, regardless of the disease onset and viral clonality. Although their VAFs were generally low, somatic mutations were detected in all polyclonal individuals (AC#1–4) using deep sequencing. These results suggested that part of the already existing clones had already acquired key somatic mutation(s) that may serve as a seed for further progression.

We further found that the mutation pattern of ACs was similar to the landscape of ATL cases^6,11^ (Fig. 6a and Supplementary Data 4). Recurrent mutations in TCR pathway genes (*PLCG1*, *VAV1*, *PRKCB*, or *CARD11*) were identified in 7 cases (70%), of which 6 were associated with clonal expansion. The sizes of major clones estimated using VIS reads (viral clonality) or VAF (mutationally defined clonality) were significantly correlated with each other in both ATL and AC (*r* = 0.86, *p* < 10^−6^, Fig. 6b), supporting the concept that somatic mutations act as major drivers of the clonal expansion of HTLV-1-infected cells from background cell populations.

Mutation tracking (follow-up period, 6–9 years) and clustering in disease progression cases (#8–10) further demonstrated that case-specific infected cells, harboring the corresponding genomic mutation(s), had clonally expanded before the disease onset (i.e., high-risk AC phase). It also appeared that the sequential acquisition of multiple mutations was associated with intratumoral diversification during disease progression (Fig. 6c, d). Although we could not perform scRNA-seq analysis directly for these cases, we detected some mutations that may have affected the transcriptome during clonal evolution in some cases. For example, a *STAT3* gain-of function mutation, analogous to ATL#3, was detected in a possible founder clone in AC#10. At the terminal acute-phase transformation, additional mutations in the TF genes, namely, *TP53*, *IRF4*, and *NOTCH1*, triggered subclonal evolution in a short period. The transcription-affecting mutations in *NOTCH1* (PEST domain mutation detected in ATL#2 and MT-1 cells) and *PRKCB* (D427N detected in ATL#3 and KOB cells) were also found in the drastic expansion of founder clones at disease onset in AC#9 and AC10, respectively. These data suggested that multiple mutation-associated transcriptomic transitions seem to be involved in the evolutionary process of clones from an early, pre-onset stage to disease progression.

### Single-cell transcriptome in HTLV-1 carriers: the cases of AC#2, #5, and #6

**Fig. 7:**
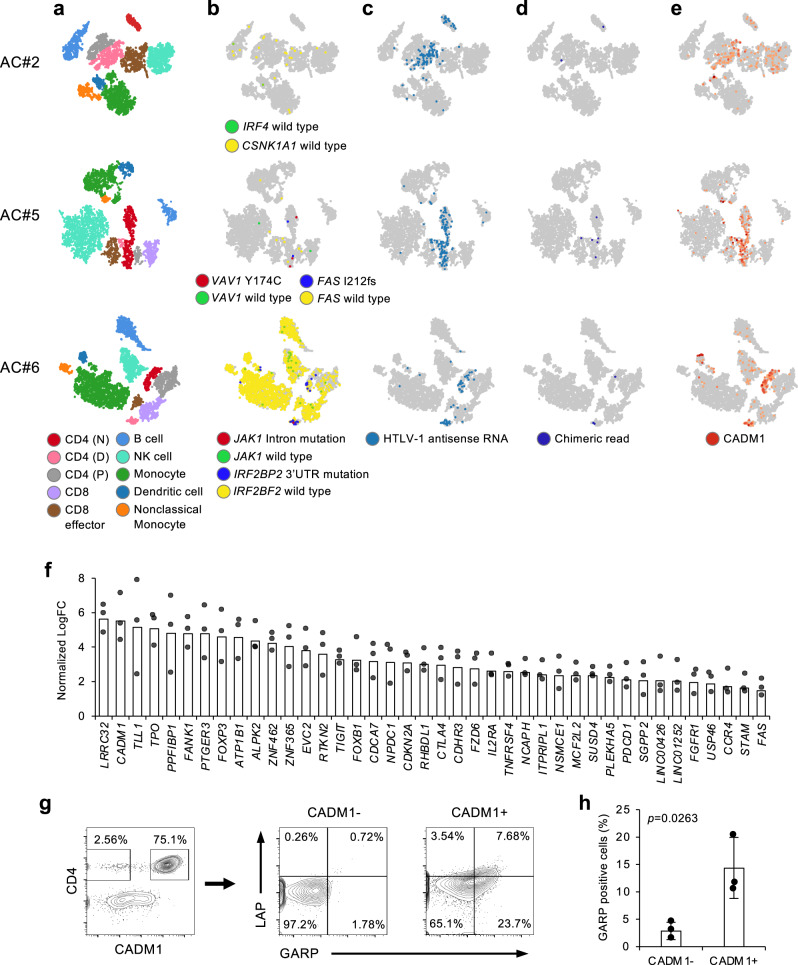
Single-cell analysis in HTLV-1-infected carriers. **a**–**e** *t*-SNE projection of scRNA-seq data in AC#2, #5, and #6, with cells colored according to lineage inference (**a**), single-cell mutation detection (**b**), viral antisense RNA (**c**), host–virus chimeric transcripts (**d**), and *CADM1* expression (**e**). **f** Common differentially upregulated 40 genes in HTLV-1-infected cells in three ACs (FC ≥ 2 vs. uninfected CD4^+^ cluster, two-sided *p* ≤ 0.05). **g** GARP and LAP staining of PBMC from ATL#3 gated on CD4^+^/CADM1^−^ and CD4^+^/CADM1^+^ subpopulations. Quadrants are set based on background staining from isotype controls. **h** Bar graph shows GARP-positive cells (%) in PBMC from three ACs (AC#2, #5, #6) gated on CD4^+^/CADM1^−^ and CD4^+^/CADM1^+^ subpopulations. mean ± SD. A two-sided Student’s *t* test was performed.

For three AC cases (AC#2, #5, and #6), fresh specimens were available, and we therefore conducted scRNA-seq analysis. Given the advantages of exploring precancerous states in terms of detectable viral infection in our platform, we analyzed the transcriptome features of virus-infected but not yet fully transformed cells (Fig. 7a–e, Supplementary Table 3, and Supplementary Data 2).

Marker gene expression was used for lineage inference (Supplementary Fig. 11). CADM1 and CD7 patterns specified HTLV-1-infected cells. The infected cell cluster showed a *CCR7*-negative memory T cell lineage. For these cells, viral antisense RNA reads and host–virus chimeric reads were also confirmed. Variant reads, representing genomic mutations, were barely detected in the corresponding cells, although the two ACs (AC#5, #6) showed a partial expansion of the infected clones.

The transcriptome of HTLV-1-infected cells was significantly different from that of uninfected T cells and cells of other lineages. We identified 40 common differentially upregulated genes in HTLV-1-infected cells compared with virus-uninfected CD4^+^ T cells (Fig. 7f, fold change (FC) ≥2, *p* ≤ 0.05). The genes included functional genes in regulatory T cell (Treg), such as *FOXP3*, *CTLA4*, *IL2RA*, *CCR4*, *TIGIT*, *PDCD1*, *RTPKN2*, *PTGER3*, and *TNFRSF4* (OX40), some of which were identified in aggressive ATL cells (Supplementary Fig. 12a). We identified *LRRC32*, which encodes glycoprotein A repetitions pre-dominant (GARP), as the topmost overexpressed gene in infected cells. This gene was also identified as one of the genes overexpressed in post-onset ATL cases (Supplementary Fig. 12b). GARP is expressed on the surface of FOXP3^+^/Helios^+^ Tregs and plays a role in Treg suppression by anchoring latent TGF-β (latency-associated peptide; LAP) on the surface of Tregs^49^. We analyzed GARP/LAP expression by flow cytometry and found that co-expression of surface GARP/LAP was significantly increased in CADM1^+^-infected cells in ATL (#2) and AC (3 cases) (Fig. 7g, h and Supplementary Fig. 12c). Thus, the HTLV-1-infected cell population containing some expanded clones may show a Treg phenotype with functional TGF-β. Further transcriptome analysis identified EZH2 overexpression and related epigenetic silencing of H3K27me3 targets in AC (Supplementary Fig. 13). These results are consistent with a previous result based on a flow cytometry-based platform^48^. Collectively, these results showed that transcriptome figures in precancerous infected cells is partly associated with ATL.

## Discussion

In this study, we developed and applied an integrative, multimodal analytical pipeline to elucidate the clone history of ATL. The customized panel encompasses the full-length HTLV-1 provirus and hundreds of host genes recurrently mutated in ATL. By focusing the sequence data on a limited number of genomic regions, it was possible to generate approximately ×2000 reads in sequence depth. The sufficient sequence depth enabled us to quantitatively analyze the VAFs and VISs to estimate the clonal structure. To validate the identification of the clonal structure and, at the same time, to analyze the transcriptome readouts of the genomic mutations, we conducted scRNA-seq analysis for the same samples. These analyses successfully detected genetic heterogeneity in patients with ATL before and after onset who were at high risk for ATL. Through chronological single-cell analyses of genotyped specimens and functional validation, this study identified possible molecular mechanisms of multistep tumorigenesis. Multiple mutations in the TCR, STAT3, and NOTCH pathways were associated with the accumulation of transcriptomic abnormalities in founder clones. The enhanced proliferative potential was commonly detected in evolved clones with different mutation patterns, suggesting that drastic hyperproliferation mediated by key factors, such as MYC, is a critical step in the latter oncogenic process.

This approach enables us to integrate different types of previously collected biological information to obtain a comprehensive view of the molecular events occurring in a patient. Our scRNA-seq data detected clone-specific chimeric host–virus reads, particularly in clonally expanded cells, which enabled us to directly estimate the transcriptome changes of identical infected clones over time during multistep tumorigenesis. Of note, short-read sequencing did not detect the entire sequences of the chimeric transcripts, which are implicated in the biological relevance^43^. Further expansion of this analysis for a larger number of samples and a functional assessment of the *cis*-perturbed transcripts associated with the viral integration sites will shed light on the diverse mutations in subclonal architecture among patients with different symptoms. Improvements in sensitivity (e.g., targeted amplification^17,18^) and read length (e.g., long-read technology^50^), as well as utilization of future applications enabling simultaneous DNA-seq and RNA-seq in depth at a single-cell resolution, should lead to the development of approaches for the early diagnosis and pre-emptive therapeutic strategy for this disease, which currently has a poor prognosis. Early detection and characterization of a minimal premalignant clone, which can develop into a fully malignant clone, may be achieved through the integration of biological and reliable genetic data to provide the shortest path for personalized care.

## Methods

### Clinical samples

Peripheral blood samples were collected from inpatients and outpatients at IMSUT Hospital, The Institute of Medical Science, The University of Tokyo. All patients with ATL were categorized into clinical subtypes, according to Shimoyama’s criteria^51^. Patients with various complications, such as autoimmune disorders and systemic infections, were excluded. Lymphoma-type patients were also excluded because ATL cells are not considered to exist in the peripheral blood of this clinical subtype. Written informed consent was obtained from all patients and asymptomatic individuals. PBMCs from ATL patients and asymptomatic HTLV-1 carriers were isolated by Ficoll separation (Ficoll-Paque, GE Healthcare). A part of genomic DNA from ATL and asymptomatic HTLV-1 carriers were also collected with informed consent as a collaborative project of the Joint Study on Predisposing Factors of ATL Development (JSPFAD). The present study was approved by the Institutional Review Board of our institute (the University of Tokyo, Tokyo, Japan). All clinical samples and corresponding experimental platforms are provided in Supplementary Table 3.

### Cell lines

The HTLV-1-infected cell line MT-2 and ATL-derived cell line MT-1 were kindly provided from an established researcher Dr. Miyoshi. ATL-derived TL-Om1 cells were kindly provided from an established researcher Dr. Sugamura. ATL-derived KOB cells were kindly provided from an established researcher Dr. Kamihira. CD3^+^ Jurkat cells were purchased from RIKEN BRC cell bank (RCB3052). The cell lines were verified by RIKEN BRC or established researchers and monitored for cross-contamination. This study authenticated the provirus integration sites and somatic mutations of the HTLV-1-infected cell lines by panel-based targeted sequencing. Cell surface expressions of CD4 and CADM1 were validated by flow cytometry. Commonly misidentified cell lines were not used in this study. The cell lines were tested for mycoplasma contamination using mycoplasma detection PCR (TAKARA, #6601) and were found to be negative for mycoplasma contamination.

### Cell culture

Normal (HTLV-1-uninfected) CD4^+^ T cells were obtained from Lonza. The HTLV-1-infected cell line MT-2, ATL-derived cell lines MT-1, TL-Om1 and KOB, and Jurkat cells were cultured in RPMI1640 (Invitrogen) with 10% of fetal bovine serum (FBS; GIBCO) and antibiotics (GIBCO). 293T and 293FT cells were cultured in Dulbecco’s Modified Eagle Medium (Nissui, Japan) with 10% of FBS and antibiotics. All cell lines and primary cultures were maintained at 37 °C with 5% CO_2_.

### Flow cytometry

HTLV-1-infected and uninfected cell populations were obtained using a HAS-flow method, as described previously^29^. Single-cell suspensions of lymphocytes were stained with fluorescent-labeled antibodies. An unlabeled CADM1 antibody (CM004-6, clone 3E1) and an isotype control chicken immunoglobulin Y (IgY) antibody (2:100) were purchased from MBL. These were biotinylated (primary amine biotinylation) using biotin *N*-hydroxysuccinimide ester (Sigma-Aldrich). Pacific Orange-conjugated anti-CD14 antibody (MHCD1430, clone TuK4) was purchased from Invitrogen. All other antibodies were obtained from BioLegend. Cells were stained using a combination of biotin-CADM1 (1:100), allophycocyanin (APC)-CD7 (clone CD7-6B7, 5:100), APC-Cy7-CD3 (clone SK7, 5:100), Pacific Blue-CD4 (clone RPA-T4, 5:100), and Pacific Orange-CD14 antibodies (5:100). APC-GARP (clone 7B11, 5:100) and FITC-LAP (TGF-β1) (clone TW7-16B4, 5:100) antibodies were used instead of CD7 antibody for GARP/LAP co-staining in CD4^+^/CADM1^−^ and CD4^+^/CADM1^+^ subpopulations. After washing, phycoerythrin-conjugated streptavidin (SA10041, 2:100, Thermo Fisher Scientific) was applied. Propidium iodide (PI; Sigma-Aldrich) was added to the samples to stain dead cells immediately before flow cytometry. A FACSAria II instrument (BD Biosciences) was used for all multicolor flow cytometry and fluorescence-activated cell sorting. PBMCs from ATL patients and HTLV-1 carriers including longitudinal samples were sorted into HTLV-1-infected cell population (CD4^+^/CADM1^+^/CD7^+^), a more aggressive cell population (CD4^+^/CADM1^+^/CD7^−^), and HTLV-1-uninfected T cells (CD4^+^/CADM1^−^/CD7^+^). TCR repertoire was screened using the Beta Mark TCR Vβ Repertoire Kit (IM3497, Beckman Coulter) and then specific clones were validated using clone-specific antibodies (Beckman Coulter). The collected data were analyzed by the FlowJo (v9.6, v10.6.1) software (Tree Star).

### Design of HTLV-1/ATL panel

To comprehensively cover genes involved in ATL, 280 human genes were selected, which included 50 genes frequently mutated in ATL^6^ and 190 genes frequently mutated in hematological and solid malignancies (Supplementary Data 1). Agilent SureDesign web-based application (https://earray.chem.agilent.com/suredesign/home.htm) was used for capture bait design. Public databases, including RefSeq, Ensembl, CCDS, Gencode, VEGA, SNP, and CytoBand, were used as references of human genome. The designed panel included 29,068 capture baits (120 bp length on average) for 280 human genes (coding exons + UTRs; 2× density tiling) and 373 capture baits (120 bp length) for HTLV-1 genome (NC_001436.1; 5× density tiling).

### Genomic analysis

Genomic DNA from enriched infected cell population, PBMCs, and cell lines were extracted using the QIAamp DNA Blood Mini Kit (QIAGEN). Normal human DNA (G1471, Promega) was used for serial dilution of cell line DNA. Target capture was conducted using the SureSelect Target Enrichment System (Agilent Technologies). The sequence data were obtained using an HiSeq2500 sequencer (Illumina) with 100-bp paired-end (PE) reads. For WGS, the TruSeq DNA Nano HT Library Prep Kit (Illumina) was used for library preparation following the manufacturer’s instruction. The libraries were sequenced by the HiSeq3000 platform (Illumina) with 100-bp PE reads. For WES, the SureSelelct XT HS Kit (Agilent Technologies) and SureSelect Human All Exon v5 (Agilent Technologies) were used for library preparation following the manufacturer’s instruction. The libraries were sequenced by the HiSeq3000 platform (Illumina) with 100-bp PE reads.

The sequenced data were aligned to the human reference genome hg38 by the BWA (v0.7.15) software. The PCR duplicates were removed using the Picard (v2.92) and SAMtools (v1.2) softwares^52^. Uninfected T cells (CD4^+^/CADM1^−^/CD7^+^ “P” population) were used as matched normal controls to call somatic mutations. The somatic mutation candidates were called using MuTect2 from the GATK (v4.0.12) software^53^ or VarScan2^54^ and annotated with ANNOVAR (v20191024)^55^. Candidate mutations with (i) ≥5 variant reads in tumor samples, (ii) a VAF in tumor samples ≥0.01, (iii) read depth ≥200, and (iv) tumor variant:normal variant ratio ≥2 were adopted and further filtered by excluding synonymous SNVs.

### Clonality analysis

The clonality analysis of HTLV-1-infected cells was performed by high-throughput sequencing-based mapping of proviral integration sites. To designate the VISs, sequence reads were aligned to human reference genome hg38 and virus genome (NC_001436.1) by BWA. PE reads spanning the viral and human genomes and soft-clipped reads (>15 bp soft-clipped region) were extracted using Perl scripts and then validated by Blastn (v2.6.0+). Clonality was calculated as population size of each clone by counting the extracted reads at host–provirus junction sites.

### Subclonal analysis based on genomic data

We used PyClone (v0.13.0) for analysis of subclonal population structure and reconstruct hierarchical trees. PyClone is based on a Bayesian clustering method, which uses a Markov chain Monte Carlo-based framework to estimate cellular prevalence values using somatic mutations. The somatic mutation candidates for PyClone were called using MuTect2, with (i) ≥5 variant reads in tumor samples, (ii) a VAF in tumor samples ≥0.05, (iii) read depth ≥200, and (iv) tumor variant:normal variant ratio ≥2. Clonal composition was investigated based on the beta binomial emission model, through which a set of clones with a discrete set of mutations (mutational clusters) were imputed together with their estimated clone size (cellular prevalence). Process of the clonal evolution were estimated by extrapolation of the estimated clone sizes at all tested timepoints. The hierarchical trees with imputed mutational subclusters were depicted by ClonEvol (v0.99.11) based on the results of clustering and cellular prevalence from the PyClone model. Integration site-based clonality was used as a parameter for clonal structure (polyclonal or monoclonal).

### RNA sequencing

Total RNA of each sample was extracted using TRIzol Reagent (Invitrogen) and quantified and qualified by Agilent 2100 Bioanalyzer (Agilent Technologies), NanoDrop (Thermo Fisher Scientific), and 1% agarose gel. Twenty ng of total RNA with RIN value above seven was used for following library preparation. Next-generation sequencing library preparations were constructed according to the manufacturer’s protocol (NEBNext UltraTM RNA Library Prep Kit for Illumina). The poly(A) mRNA isolation was performed using rRNA depletion (NEBNext H/M/R) and NEBNext Ultra II Directional RNA Kit. The mRNA fragmentation and priming were performed using NEBNext First Strand Synthesis Reaction Buffer and NEBNext Random Primers. First-strand cDNA was synthesized using ProtoScript II Reverse Transcriptase and the second-strand cDNA was synthesized using Second Strand Synthesis Enzyme Mix. The purified double-stranded cDNA (by AxyPrep Mag PCR Clean-up (Axygen) was then treated with End Prep Enzyme Mix to repair both ends and add a dA-tailing in one reaction, followed by a T-A ligation to add adapters to both ends. Size selection of Adapter-ligated DNA was then performed using AxyPrep Mag PCR Clean-up (Axygen), and fragments of ~360 bp (with the approximate insert size of 300 bp) were recovered. Each sample was then amplified by PCR for 11 cycles using P5 and P7 primers, with both primers carrying sequences that can anneal with flow cell to perform bridge PCR and P7 primer carrying a six-base index allowing for multiplexing. The PCR products were cleaned up using AxyPrep Mag PCR Clean-up (Axygen), validated using an Agilent 2100 Bioanalyzer, and quantified by Qubit 2.0 Fluorometer (Invitrogen). Then libraries with different indices were multiplexed and loaded on an Illumina HiSeq instrument according to the manufacturer’s instructions (Illumina). Sequencing was carried out using a 2 × 150 bp PE configuration; image analysis and base calling were conducted by the HiSeq Control Software (HCS) +;OLB + GAPipeline-1.6 (Illumina) on the HiSeq instrument. The sequences were processed and analyzed by GENEWIZ. For quality control, to remove technical sequences, including adapters, PCR primers, or fragments thereof, and quality of bases <20, pass filter data of fastq format were processed by Trimmomatic (v0.30) to be high-quality clean data. For mapping, Hisat2 (v2.0.1) was used to index the reference genome sequence. Finally, clean data were aligned to the reference genome via software Hisat2.

### Single-cell RNA sequencing

The scRNA-seq library was constructed by using the Chromium Controller and Chromium Single Cell 5′ Reagent Kits and 3′ Reagent Kits v2 (10× Genomics) following the standard manufacturer’s protocols. To collect live cells for scRNA-seq, PBMC cryovials [1–10 × 10^6^ cells/1 mL of CELLBANKER 1 (ZENOAQ resource)] were removed from liquid nitrogen or −80 °C freezer and warmed in a 37 °C water bath. Cells were then pelleted by centrifugation at 500 × *g* for 5 min and resuspended in phosphate-buffered saline (PBS). After twice washing with PBS, cells were then pipetted through a 40-μm filter to remove cell doublets and contamination. Cell viability (>60%) was confirmed by trypan blue staining. The collected single-cell suspension from PBMCs or HAS-flow sorted PI-negative live cell subpopulations (1.6 × 10^4^ live cells/sample) were immediately loaded onto the 10× Chromium controller in an effort to recover thousands of cells from each subpopulation for library preparation and sequencing.

Gel beads were prepared according to the standard manufacturer’s protocols. Oil partitions of single cell with oligo-coated gel beads were captured and reverse transcription was performed, resulting in cDNA tagged with a cell barcode and unique molecular index (UMI). The library was sequenced using the HiSeq3000 system (Illumina) according to the manufacturer’s instruction. Sequencing was carried out using a 1 × 91–98 bp single-end configuration (default setting), which is sufficient to align confidentially to the transcriptome. For cases ATL#2 and #3, additional sequencing was carried out using a 2 × 150 bp PE configuration to enhance variant detection efficiency. After sequencing analysis, fastq files were created by the Cell Ranger ver3.1.0 mkfastq pipeline (10× Genomics). The obtained fastq files were mapped to the reference genome provided by 10× Genomics (GRCh38). Cell Ranger count pipeline (v3.1.0) was used to perform demultiplexing, aligning reads, filtering, clustering, and gene expression analyses, using default parameters. Briefly, after read trimming, Cell Ranger used an aligner called STAR, which performs splicing-aware alignment of reads to the genome. Cell Ranger further aligned exonic and intronic confidently mapped reads to annotated transcripts by examining their compatibility with the transcriptome. Only uniquely mapping exonic reads were carried forward to UMI counting. After the UMI filtering steps with default parameters and expected cell counts, each observed barcode, UMI, gene combination was recorded as a UMI count in the feature-barcode matrix. The workflow also performed an improved Calling Cell Barcodes algorithm and identified the primary mode of high RNA content cells and also captured low RNA content cells.

After data processing, we recovered quality-assured data for secondary analysis of gene expression. For example, we recovered 5251 cells (D population) and 7163 cells (N population) with median UMI counts of 4752 per cell (D population) and 1880 per cell (N population) in scRNA-seq analysis for ATL#6 T2. To correct batch effects between timepoints, we used a Cell Ranger merge algorithm. To regress out the cell–cell variation in gene expression driven by batch and cluster data with corrected data in T1/T2 scRNA-seq data (ATL#3), we used standard Seurat v3 integration workflow with functions FindIntegrationAnchors() and IntegrateData(). The Cell Ranger data or batch-corrected data were imported into the Loupe Cell Browser Software (v4.2.0) for *t*-distributed stochastic neighbor embedding (*t*-SNE)-based clustering, heatmap generation, and gene expression distribution plots.

### Single-cell mutation identification and analysis

RNA variants from scRNA-seq data were validated from curated BAM files based on the results of Cell Ranger. For each cell barcode in the filtered Cell Ranger barcode list and each somatic variant in the targeted sequencing data, variant bases were identified. Only reads that had both a Chromium Cellular Barcode (CB) tag and a Chromium Molecular Barcode tag were included. We then obtained the cell-associated tag for downstream analysis of UMIs. CB tags with the variant reads extracted by SAMtools were defined as at least one mutant read detected and mapped on each *t*-SNE projection using the Loupe Cell Browser Software. Almost all variants were validated by manual review to accurately identify mutant cells. One-sided Fisher exact tests were used to identify cell clusters that were enriched for somatic mutations (*p* ≤ 0.05).

### Virus reads and host-virus chimeric reads from single-cell data

For detection of virus reads, we processed Cell Ranger GRCh38-aligned sequence data. No-map and soft-clipped reads (>20 bp soft-clipped) were extracted using Python scripts. The pass filter data of fastq format were processed to remove adapter and polyA sequences. The high-quality clean data were then aligned to human reference genome (hg38) and virus genome (NC_001436.1) via software STAR. For detection of cells expressing virus genes, CB tags with virus reads were defined as at least one virus read detected. Almost all virus-aligned reads were derived from antisense strand. Both host- and virus-aligned soft-clipped reads were extracted as host–virus chimeric reads. Genomic breakpoints of chimeric reads were analyzed from supplementarily mapped data from STAR alignment to link the clone-specific chimeric reads with the VISs identified in the corresponding clones. The extracted CB tags with virus antisense reads or clone-specific host–virus chimeric reads were mapped on *t*-SNE projection using Loupe Cell Browser. One-sided Fisher exact tests were used to identify cell clusters that were enriched for virus reads (*p* ≤ 0.05).

### Cluster assignment and single-cell expression analysis

Expression patterns of *CD4*, *CADM1*, and *CD7* were used and overlaid on the *t*-SNE to identify HTLV-1-infected subpopulations. CBs with HTLV-1-derived antisense transcripts were also overlaid on the *t*-SNE. Stable expression of HTLV-1 antisense RNA (predominantly *HBZ*) served for inference of infected cells (*p* ≤ 0.05). Infected clone-specific host–virus chimeric reads were significantly enriched in each cluster (*p* ≤ 0.05). To detect the mutation-harboring clones estimated by PyClone, RNA variants from scRNA-seq data were validated from curated BAM files based on the results of Cell Ranger. CB tags with variant reads were defined as at least one mutant read detected and mapped on each *t*-SNE projection (*p* ≤ 0.05).

For analysis of scRNA-seq from PBMCs, major cell types were annotated by marker genes, including CD4^+^ T cell (*CD3D*^+^, *CD4*^+^), CD8^+^ T cell (*CD3D*^+^, *CD8B*^+^), CD8^+^ effector CTL (*CD3D*^+^, *CD8B*^+^, *KLRB1*^+^, *CCR7*^−^), NK cell [*NCAM1*^+^ (CD56)], B cell (*CD79A*^+^, *CD19*^+^), monocyte (*CD14*^+^), nonclassical monocyte [*FCGR3A*^+^ (CD16)], and dendritic cell (*CD1C*^+^). The assigned lineage clusters were mapped on *t*-SNE projection using Loupe Cell Browser.

Log_2_ FC and median-normalized average values of assigned clusters were obtained via Loupe Cell Browser and used in the following analysis of differentially expressed genes within each cluster. Manual clustering based on expression patterns was curated by original Python scripts or polygonal selection tool (Loupe Cell Browser interface).

### Bioinformatic analysis and statistics

IGV tool^56^ was used for visualizing and interpreting the results of DNA-seq and RNA-seq. For differentially expressed gene analysis, HTSeq (v0.6.1) estimated gene and converted read counts to transcripts per million (TPM) from the PE clean data. Differentially expressed genes were selected based on the absolute Log_2_ FC of ≥1. Selected genes were subjected to the hierarchical clustering analysis using iDEP.91 pipeline that contains DESeq2 package^57^. GSEA^58^ was performed using the GSEA software (v4.0.3) (http://www.broadinstitute.org/gsea) with 1000 permutations. Gene sets used in this study were selected from the MSigDB hallmark gene sets (http://www.broadinstitute.org/gsea/msigdb/collections.jsp). Significantly enriched gene sets were evaluated by normalized enrichment score and nominal *p* value (*p* ≤ 0.001). Gene Ontology analysis was performed by DAVID Bioinformatics Resources (https://david.ncifcrf.gov/). For target gene analysis of NOTCH1, STAT3, RBPJ, and NFAT, enrichment data and bigwig format from previous ChIP-seq studies were obtained from the ChIP-Atlas database (https://chip-atlas.org/)^59^. Gene lists of NOTCH1 and STAT3 targets were created by integrating of hallmark genes and ChIP-bound genes. Significant differences in gene expression and other biological assays between the two groups were analyzed by a two-sided Student’s *t* test. Adjustments were not made for multiple comparisons. Correlations between two groups were analyzed by a two-sided Pearson’s correlation coefficients and probabilities of overlap between gene sets were statistically tested.

### Data visualization

Box plots, beeswarm plots, hierarchical clustering, and correlation matrix were analyzed and visualized by using R (v3.2.3). Box plots are defined as follows: the middle line corresponds to the median; lower and upper hinges correspond to first and third quartiles. The upper whisker extends from the hinge to the largest value no further than 1.5 × IQR from the hinge (where IQR is the inter-quartile range or distance between the first and third quartiles). The lower whisker extends from the hinge to the smallest value at most 1.5 × IQR of the hinge. All data points are overlaid on the box plot.

### Quantification of HTLV-1 PVL

Measurement of HTLV-1 PVL of PBMC samples was described previously^33^. Briefly, quantitative multiplex real-time PCR was performed with two sets of primers specific for the HTLV-1 provirus and the human gene encoding the RNase P enzyme. The PVL was expressed as copy numbers per 100 PBMCs, based on the assumption that infected cells harbored one copy of the integrated HTLV-1 provirus per cell.

### Functional analysis of the NOTCH1 pathway

For quantification of NOTCH1 target genes, DNaseI-treated total RNA was subjected to reverse-transcriptase (RT) reaction using ReverTra Ace qRT-PCR Master Mix (TOYOBO) with the manufacturer’s protocol. Random primer-based synthesized cDNA was analyzed by quantitative PCR using a real-time PCR system (Thermal cycler Dice, TAKARA). The specific PCR was performed using gene-specific primers (Supplementary Table 4) and SYBRGreen (SYBR Select Master Mix, Applied Biosystems). The level of *RPL19* mRNA was also quantified for internal control.

Protein levels of ICN1 and Cbl-b were analyzed by immunoblotting with primary antibodies, as follows: ICN1 (Cleaved Notch1 (V1754), #4147, 1:1000, Cell Signaling Technology), Cbl-b (sc-8006, 1:200, Santa Cruz), and β-actin (sc-69879, 1:1000, Santa Cruz). Alkaline phosphatase-conjugated anti-mouse (S3721, 1:2000, Promega) and anti-rabbit (S3731, 1:2000, Promega) secondary antibodies and BCIP/NBT substrate (S3771, Promega) were used for detection. The uncropped scans of the blots are provided in the Source data file.

For evaluation of the anti-growth activity of GSI, 5 × 10^4^ cells of MT-1 and TL-Om1 were plated in 12-well flat bottom plate with optimized media with 10% of FBS and simultaneously treated with the indicated does of GSI (DAPT from Cayman) solution in dimethyl sulfoxide for 6 days. The cells were maintained by passage into fresh medium at day 3. The cell numbers were evaluated by the Cell Counting Kit-8 (Dojindo) following the manufacturer’s protocol.

### Functional analysis of VAV1 on the TCR pathway

Point mutagenesis for mimicking the mutations of *VAV1* was accomplished with the PrimeSTAR Mutagenesis Basal Kit (TAKARA) and specific primer sets (Supplementary Table 4). Established mutant plasmids were validated by sanger sequencing. For stable expression of VAV1, the C-terminal hemagglutinin (HA)-tagged VAV1 series (VAV1^WT^, VAV1^Y174C^, and VAV1^M501R^) were cloned into a CSII-EF1α-IRES-Venus replication-defective, self-inactivating lentivirus vector (RIKEN, BRC, Japan). The established viral vectors were co-transfected with the packaging plasmid (pCAG-HIVgp) and the VSV-G- and Rev-expressing plasmid (pCMV-VSV-G-RSV-Rev) into 293FT cells. High-titer viral solutions were prepared by centrifugation-based concentration and used for transduction into Jurkat cells. The infection was attained by spinoculation method (1800 r.p.m., 2 h) and then cultured in an appropriate condition for 3 days. After cultivation, Venus-positive cell populations were confirmed and then sorted by fluorescence-activated cell sorting (FACS). VAV1 expression was validated by immunoblotting with antibodies against VAV1 (#4657, 1:1000, Cell Signaling Technology) and HA (code 561, 1:1000, MBL).

For analysis of cellular NFAT activity, Jurkat cells stably expressing VAV1 series were transfected with pGL3 vector containing an NFAT-binding domain upstream of a luciferase reporter (#17870 from Addgene) using Lipofectamine 2000 (Thermo Fisher Scientific). The transfectants were seeded in 48-well plates and stimulated with or without 1 μg/mL plate-immobilized anti-CD3 monoclonal antibody (130-093-387, Miltenyi Biotec) for 20 h. Cells were recovered and then firefly and Renilla luciferase activities were measured using a Dual-Luciferase Reporter Assay (Promega) according to the manufacturer’s protocol.

### Functional analysis of PKCβ

Short hairpin RNA (shRNA)-mediated knockdown was described previously^48^. Briefly, shRNA-encoding oligonucleotides (Supplementary Table 4) were cloned into a CS-RfA-EVBsd vector via pENTR4-H1. The established viral vectors were co-transfected with packaging plasmids into 293FT cells. High-titer viral solutions were used for transduction into KOB cell lines. shRNA-expressing Venus^+^ cell populations were sorted by FACS (Venus competition assay). Gene expression levels were analyzed by qRT-PCR with specific primer sets (Supplementary Table 4).

### Reporting summary

Further information on research design is available in the Nature Research Reporting Summary linked to this article.

## Supplementary information

Supplementary Information

Description of Additional Supplementary Files

Supplementary Data 1

Supplementary Data 2

Supplementary Data 3

Supplementary Data 4

Reporting Summary

## Data Availability

All raw sequencing data for Target-seq, RNA-seq, and scRNA-seq have been deposited in the National Bioscience Database Center (NBDC) Human Database, which is associated with DNA DataBank of Japan (DDBJ) under an accession number JGAS000301. The data are available under restricted access; access can be obtained by completing the NBDC application form. The publicly available gene expression data used in this study are available in the Gene Expression Omnibus (GEO) database under accession code GSE13738. The ChIP-seq publicly available data used in this study are available in the ChIP-Atlas database under accession codes NOTCH1; SRX070882, RBPJ; SRX070884, STAT3; SRX5801455, and NFATC2; SRX3279752. The remaining data are available within the article, Supplementary Information, or Source data file. Source data are provided with this paper.

## Source data

Source Data
