# Supplementary material for: Chronological genome and single-cell transcriptome integration characterizes the evolutionary process of adult T cell leukemia-lymphoma
Source: Nat Commun. 2021 Aug 10;12:4821. doi: 10.1038/s41467-021-25101-9 (PMC8355240; doi:10.1038/s41467-021-25101-9)
Supplement: Supplementary file 1 — Supplementary Information [file 41467_2021_25101_MOESM1_ESM.pdf]

Supplementary Information

Chronological genome and single-cell transcriptome integration characterizes the evolutionary process of adult T cell leukemia-lymphoma

Makoto Yamagishi[1], Miyuki Kubokawa[1], Yuta Kuze[2], Ayako Suzuki[3], Akari Yokomizo[1], Seiichiro Kobayashi[4], Makoto Nakashima[1], Junya Makiyama[5], Masako Iwanaga[6], Takahiro Fukuda[7], Toshiki Watanabe[8], Yutaka Suzuki[2]*, Kaoru Uchimaru[1]*

[1]Laboratory of Tumor Cell Biology, Department of Computational Biology and Medical Sciences, Graduate School of Frontier Sciences, The University of Tokyo, Tokyo, Japan
[2]Laboratory of Systems Genomics, Department of Computational Biology and Medical Sciences, Graduate School of Frontier Sciences, The University of Tokyo, Tokyo, Japan
[3]Laboratory of Multi-Omics Data Analysis, Department of Computational Biology and Medical Sciences, Graduate School of Frontier Sciences, The University of Tokyo, Tokyo, Japan
[4]Department of Hematology, Kanto Rosai Hospital, Kanagawa, Japan
[5]Department of Hematology, Sasebo City General Hospital, Nagasaki, Japan.
[6]Department of Clinical Epidemiology, Nagasaki University Graduate School of Biomedical Sciences, Nagasaki, Japan.
[7]Department of Hematopoietic Stem Cell Transplantation, National Cancer Center Hospital, Tokyo, Japan.
[8]Department of Practical Management of Medical Information, Graduate School of Medicine, St. Marianna University, Kanagawa, Japan.

*Correspondence: uchimaru@edu.k.u-tokyo.ac.jp (K.U.); ysuzuki@edu.k.u-tokyo.ac.jp (Y.S.)

Supplementary Fig. 1

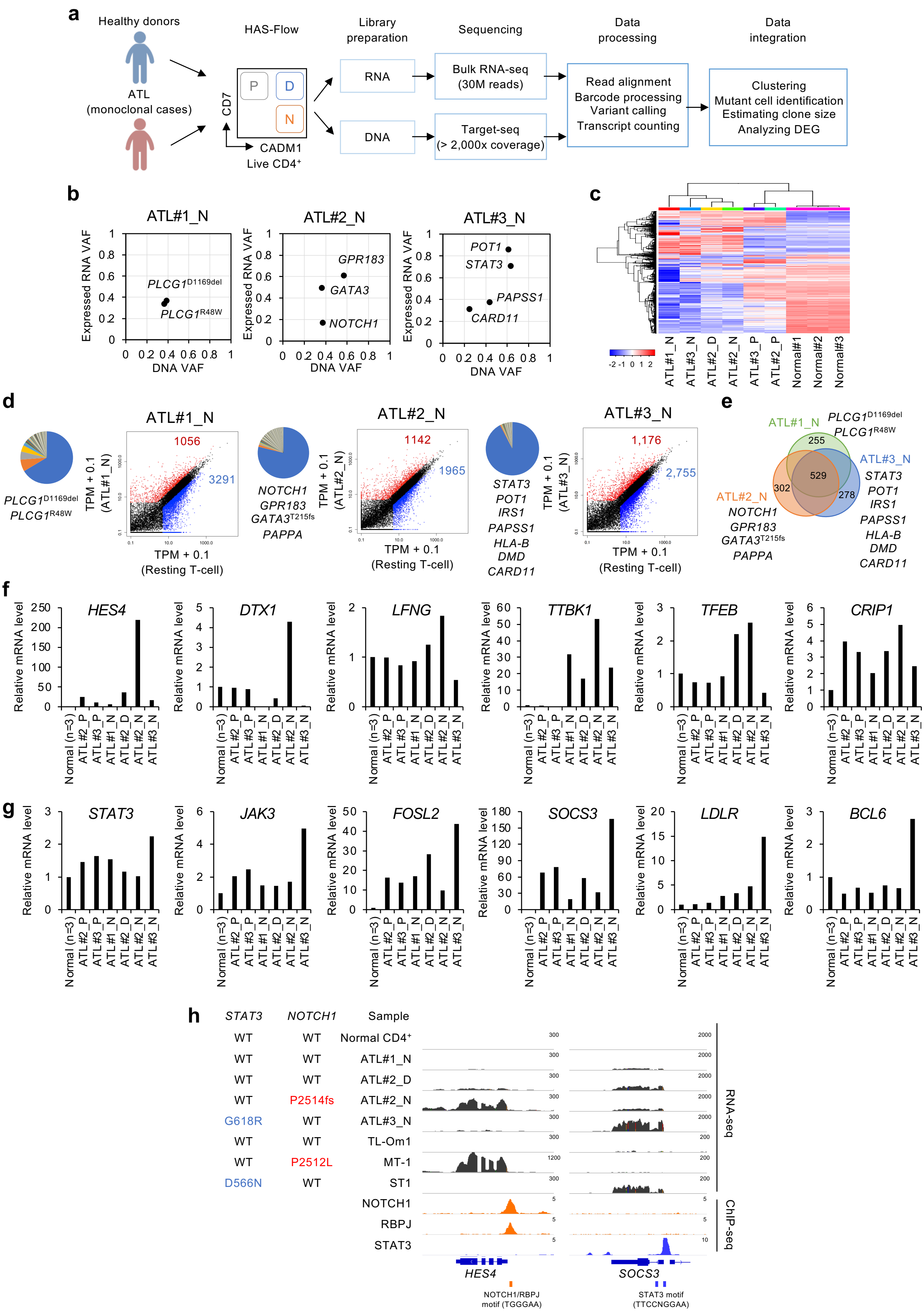

Supplementary Fig. 1 Deep RNA-seq analysis of genotyped monoclonal ATL cases.
a Workflow shows the collection and processing of fresh peripheral blood samples from ATL patients by HAS-flow, followed DNA and RNA sequencing platform.
b Deep RNA-seq analysis (30M reads/sample) for $CD4^{+}$/$CADM1^{+}$/$CD7^{-}$ (N) populations in ATL#1~3. Scatter plots show relationships between VAF values of DNA (x-axis) and expressed RNA (y-axis). Low-expressed RNAs are omitted.
c Heatmap and dendrogram show result of hierarchical clustering based on most variable 3,000 genes.
d Pie charts show VIS reads-based clonality with mutated genes. Scatter plots show Transcripts Per Kilobase Million (TPM) + 0.1 values from RNA-seq data as compared with uninfected normal $CD4^{+}$ T-cells (averaged TPM from n = 3). Upregulated (red) and downregulated (blue) genes are colored.
e Venn diagram depicts overlap between upregulated genes (FC ≥ 2 vs. normal $CD4^{+}$ T-cells).
f, g Bar graphs show relative mRNA levels of NOTCH1 (**f**) and STAT3 (**g**) target genes in normal $CD4^{+}$ T-cells (n = 3) and HAS-flow subpopulations from clinical samples. Target genes of NOTCH1 and STAT3 were selected by integrating of hallmark genes and ChIP bound genes. Relative data were analyzed from RNA-seq TPM values (normal $CD4^{+}$ T-cells = 1).
h IGV images depict levels of mapped RNA (RNA-seq) in clinical samples and cell lines and chromatin-bound NOTCH1, RBPJ (ChIP-seq data from CUTLL1 T-cell leukemia cells), and STAT3 (from normal Th1 cells) at *HES4* and *SOCS3* genes.

Supplementary Fig. 2

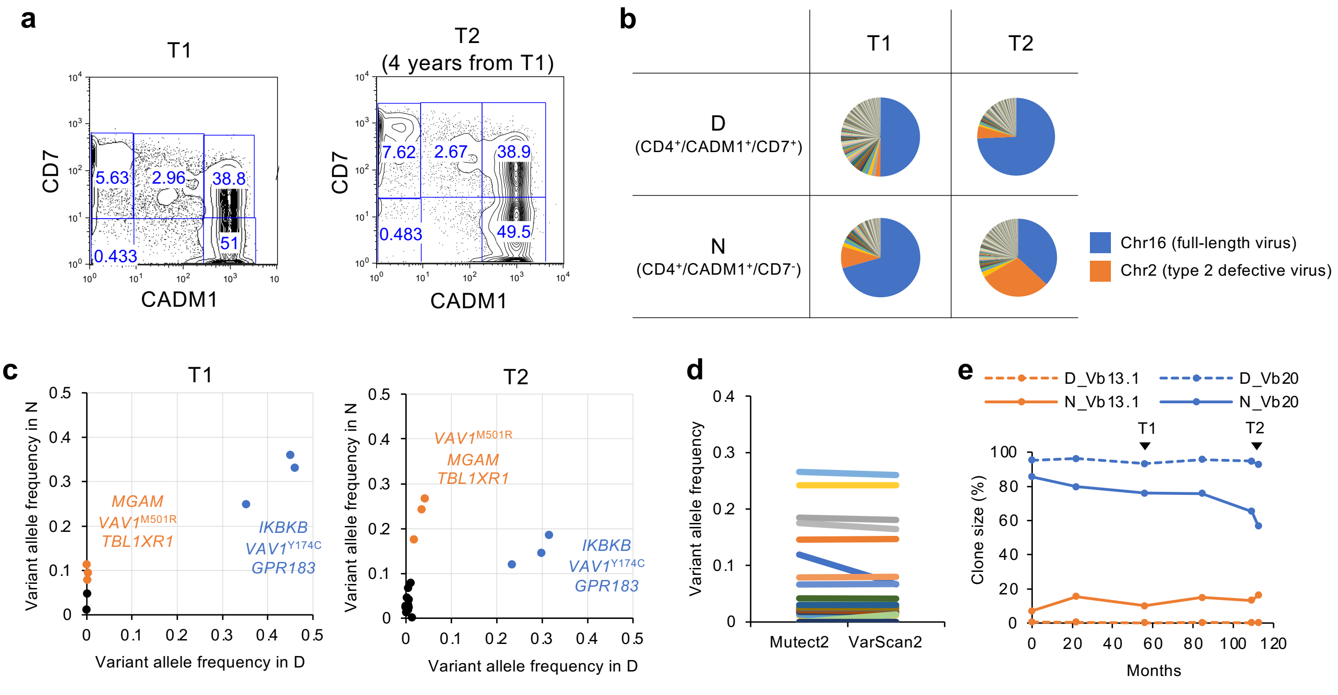

f

ID	Phenotype	Integration site	Provirus	Clone size in N (Timepoint 1)	Clone size in N (Timepoint 2)	Somatic mutation	TCR clone
Clone A	D, N	Chr16	Full-length	67.0%	37.0%	*VAV1* (Y174C)<br>*IKBKB* (E247K)<br>*GPR183* (F306fs)	Vb20
Clone B	N	Chr2	Type 2 defective	8.50%	29.7%	*VAV1* (M501R/V)<br>*MGAM* (V2121A)<br>*TBL1XR1* (P305del)	Vb13.1

Supplementary Fig. 2 Longitudinal clonality analysis in an indolent ATL case (ATL#6).
a HAS-flow plots represent CADM1/CD7 pattern within $CD4^{+}$ lymphocytes from peripheral blood at T1 and T2 (4 years later) in smoldering ATL case (ATL#6).
b Pie charts show clonalities of each D and N subpopulation based on VIS reads at T1 and T2.
c Scatter plots show VAF values of mutated genes in D (x-axis) and N (y-axis) at T1 and T2. Putative driver genes in Clone A (blue) and Clone B (orange) are annotated.
d Line chart shows results of somatic mutation calling by Mutect2 and VarScan2. Synonymous mutations are omitted.
e Longitudinal TCR repertoire analysis. Gradual expansion of T-cell clone expressing TCR Vβ13.1 were detected for approximately 10 years. Arrowheads indicate time points when deep sequencing was conducted.
f Estimated properties of Clone A and Clone B.

Supplementary Fig. 3

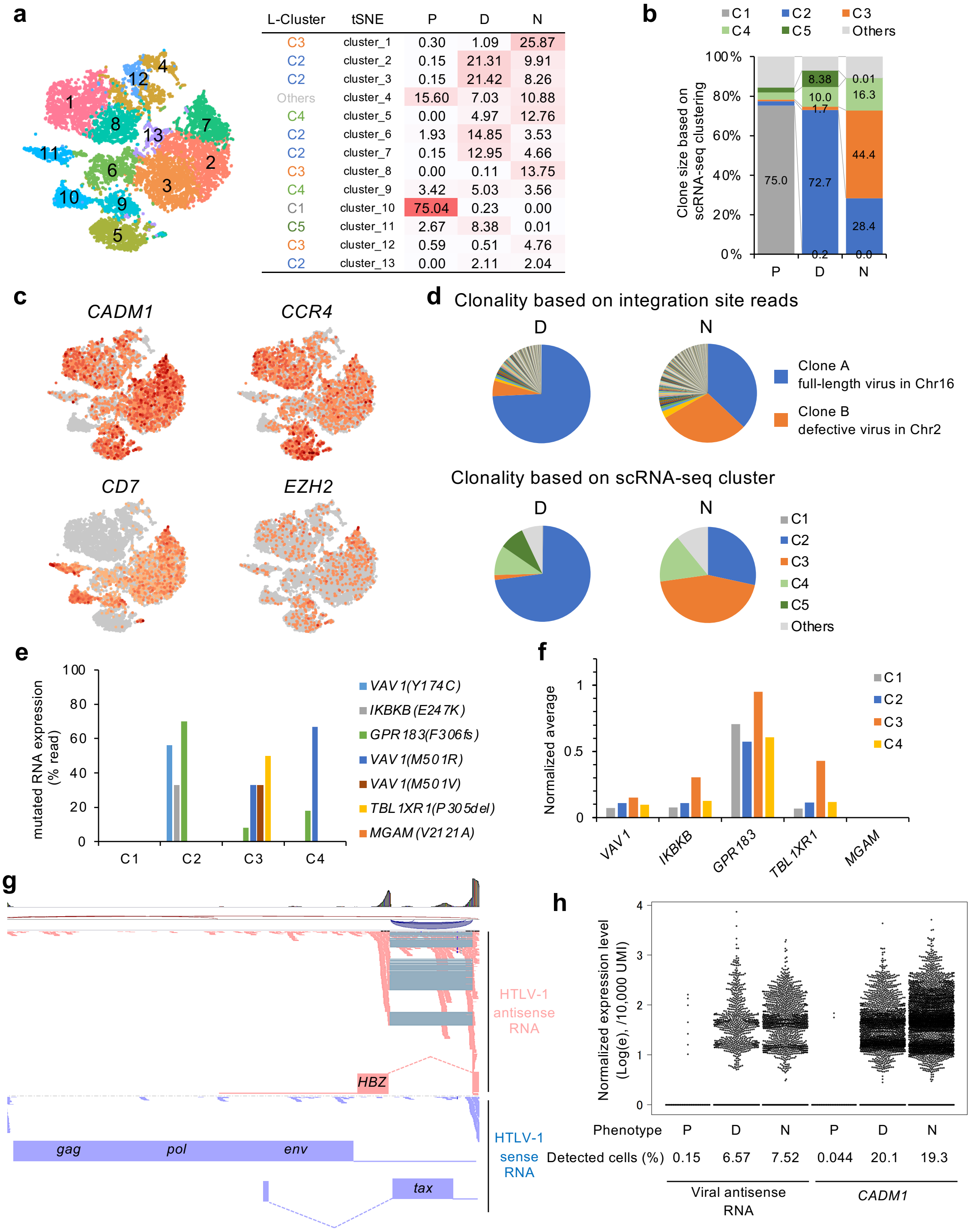

L-Cluster	tSNE	P	D	N
C3	cluster_1	0.30	1.09	25.87
C2	cluster_2	0.15	21.31	9.91
C2	cluster_3	0.15	21.42	8.26
Others	cluster_4	15.60	7.03	10.88
C4	cluster_5	0.00	4.97	12.76
C2	cluster_6	1.93	14.85	3.53
C2	cluster_7	0.15	12.95	4.66
C3	cluster_8	0.00	0.11	13.75
C4	cluster_9	3.42	5.03	3.56
C1	cluster_10	75.04	0.23	0.00
C5	cluster_11	2.67	8.38	0.01
C3	cluster_12	0.59	0.51	4.76
C2	cluster_13	0.00	2.11	2.04

Phenotype	P	D	N	P	D	N
Detected cells (%)	0.15	6.57	7.52	0.044	20.1	19.3
	Viral antisense RNA			CADM1		

Supplementary Fig. 3 Clustering of subclonal populations by scRNA-seq in ATL#6.

a t-distributed stochastic neighbor embedding (t-SNE) projection from P, D, N subpopulations (total 13,087 cells) reveals 13 distinct graph-based clusters at T2. Right table summarizes large clusters (C1 - C5) and cell proportion (%).

b Stacked bar graph shows estimated clone sizes in P, D, N cells based on population frequencies of large clusters.

c t-SNE plots show expression levels of marker genes. Cells are colored according to expression level of specific marker genes (upregulation of *CADM1*, *CCR4*, *EZH2*; downregulation of *CD7*).

d Pie charts show clonalities of D and N populations based on VIS reads (upper panels) or t-SNE-based clustering (lower panels).

e Bar graph shows mutated RNA expression (variant reads %) in each large cluster from scRNA-seq data.

f Bar graph shows the normalized average of the mutated genes. *MGAM* RNA was not detected.

g IGV image depicts HTLV-1 antisense RNA (top) and sense RNA (bottom) from scRNA-seq data (1 × 91~98bp single-end reads).

h Expression levels of HTLV-1 antisense RNA and *CADM1* detected in scRNA-seq libraries from P, D, and N subpopulations. Detected cells (%) from total cell numbers are provided.

Supplementary Fig. 4

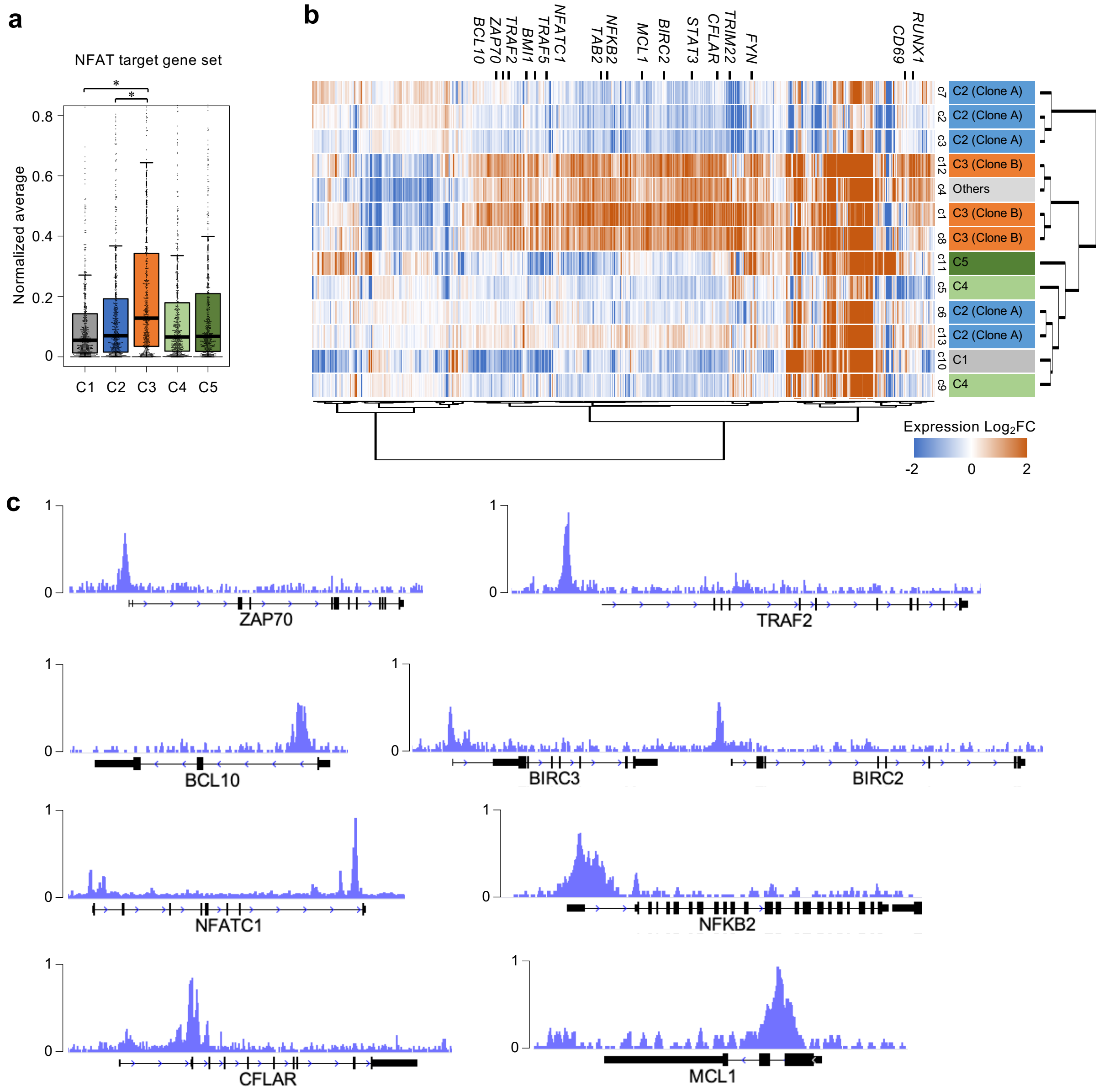

Supplementary Fig. 4 Regulation of NFAT activity within subclonal populations in ATL#6.
a Box plots show normalized average of expression levels of NFAT target genes (167 genes from GSE13738 dataset). NFAT target genes were defined by ChIP-Atlas data (SRX3279752, peak values ≥ 50). *p* values are tested as Log_2 fold changes. The middle lines within box plots correspond to the medians; lower and upper hinges correspond to first and third quartiles. The upper whisker extends from the hinge to the largest value no further than 1.5 * IQR. The lower whisker extends from the hinge to the smallest value at most 1.5 * IQR. All data points are overlaid on the boxplot. * $p \leq 0.01$ (two-sided Student's *t*-test).
b Clustered heatmap depicts expression levels of NFAT target genes.
c IGV images depict bound NFAT profiles at functionally important genes in $CD4^+$ T-cells. ChIP-seq bigwig data were imported from ChIP-Atlas database.

Supplementary Fig. 5

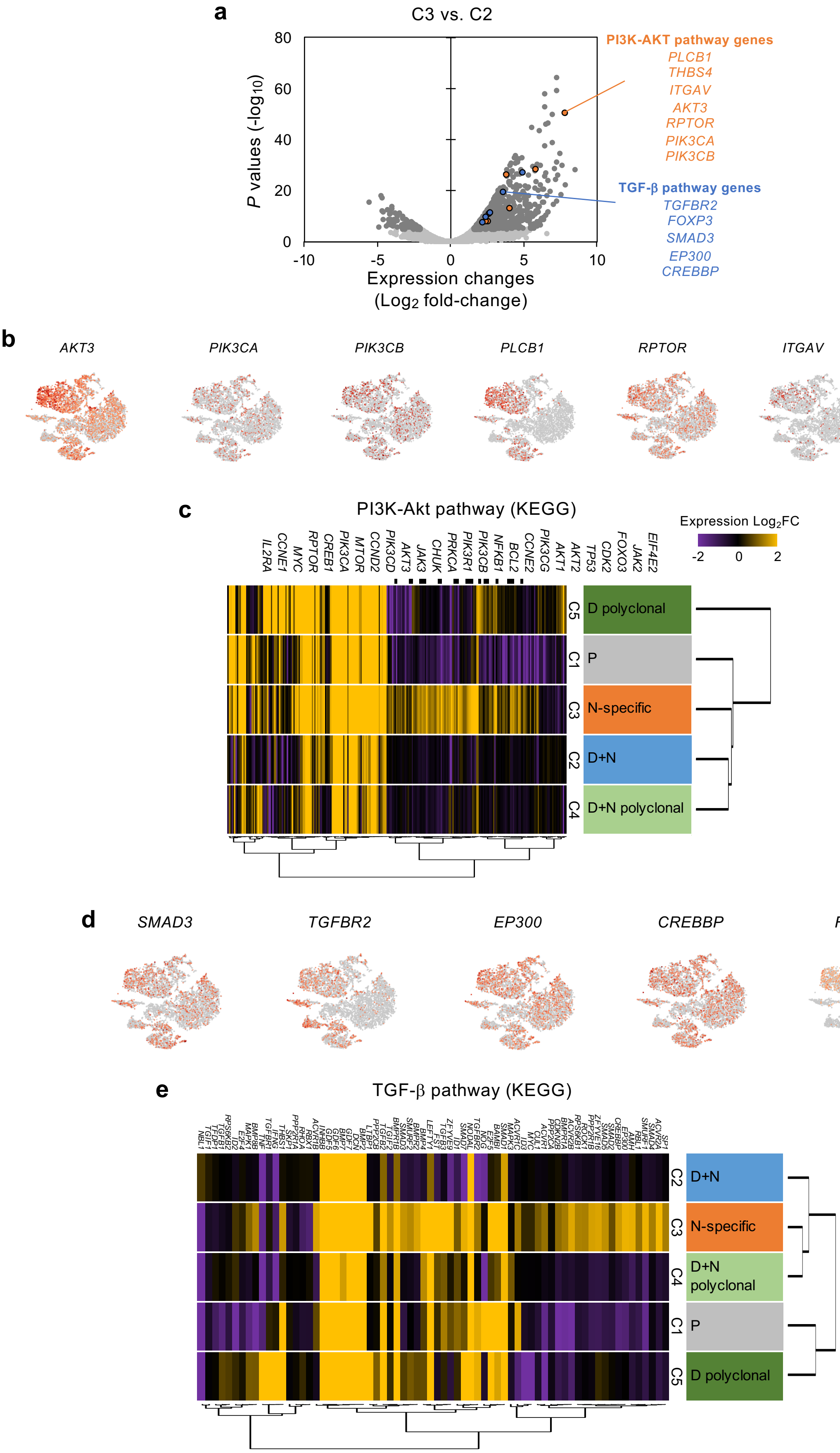

Supplementary Fig. 5 Evaluation of signaling pathways by scRNA-seq data in ATL#6.
a Volcano plot shows differentially expressed genes in C3 cluster in ATL#6. Differential expression is shown as Log_2 fold change and is plotted against $-Log_{10}(p$ value) (two-sided Student's t-test). Dark gray plots represent differentially expressed genes [Abs(FC) ≥ 2, $p \leq 10^{-5}$]. Representative pathway components are indicated by colors.
b t-SNE plots of ATL#6 show expression levels of genes involved in PI3K-AKT pathway (KEGG). Cells are colored according to expression level of indicated genes.
c Clustered heatmap depicts expression levels of genes involved in PI3K-AKT pathway.
d t-SNE plots of ATL#6 show expression levels of genes involved in TGF-β pathway (KEGG). Cells are colored according to the expression level of indicated genes.
e Clustered heatmap depicts expression levels of genes involved in TGF-β pathway.

Supplementary Fig. 6

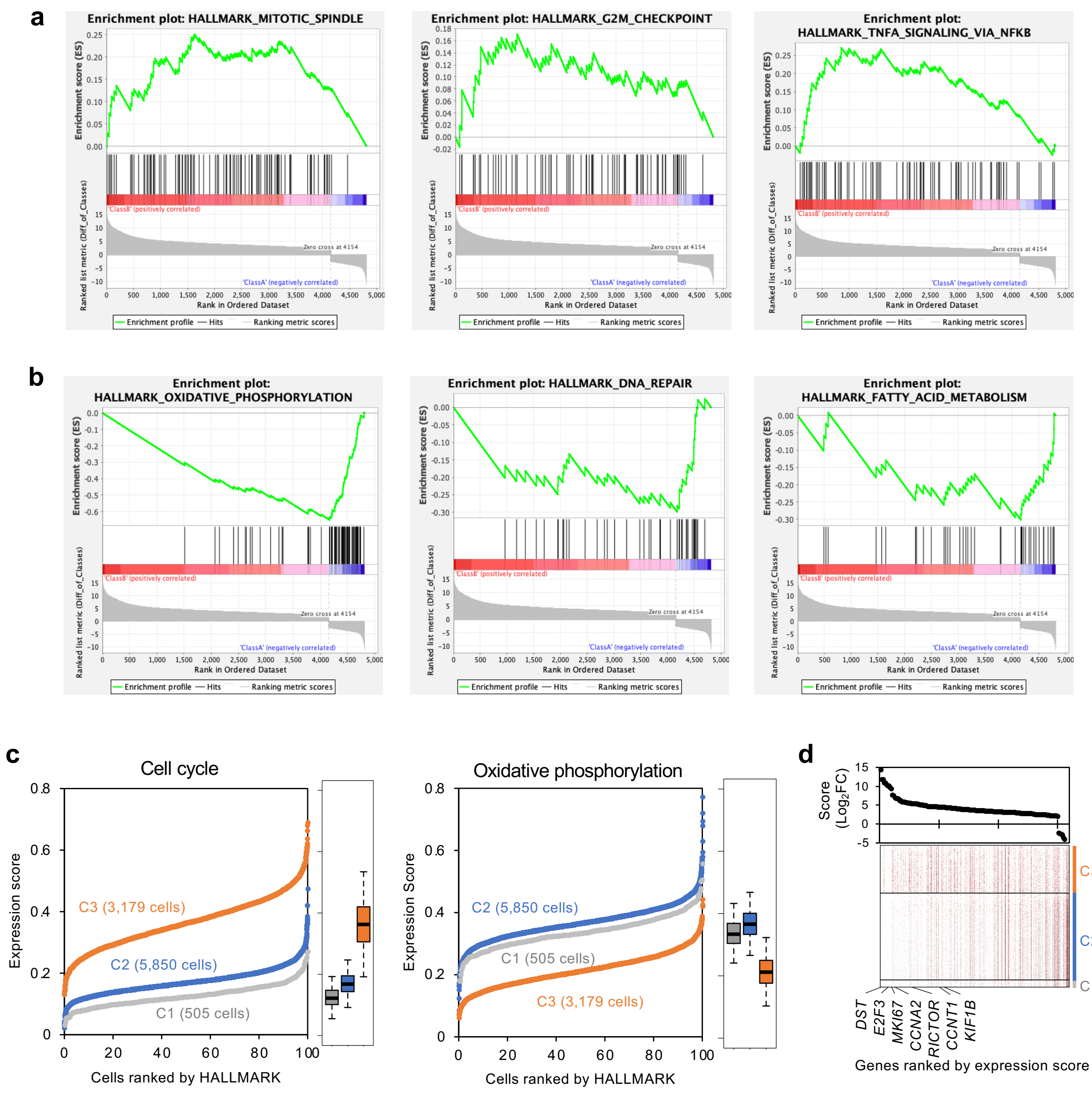

Supplementary Fig. 6 Gene set enrichment analysis of scRNA-seq data in ATL#6.

a, b Gene set enrichment analysis (GSEA) on C3 cluster cells (Clone B) (**a**) and C2 cluster cells (Clone A) (**b**) in ATL#6. Statistically significant 5,106 genes (two-sided $p \leq 0.01$) were used as input data. Gene sets enriched in Clone B ($p \leq 0.05$) are involved in mitotic cell proliferation and NF-κB signaling; Gene sets enriched in Clone A are involved in oxidative phosphorylation and DNA repair.

c Scatter plots show cells ranked by expression score of cell cycle gene set (HALLMARK_G2M_CHECKPOINT and HALLMARK_MITOTIC_SPINDLE) (left) and oxidative phosphorylation gene set (HALLMARK_OXIDATIVE_PHOSPHORYLATION) (right) in clusters C1 (505 cells), C2 (5,850 cells), and C3 (3,179 cells). Box plots show distribution of the score. The middle lines within box plots correspond to the medians; lower and upper hinges correspond to first and third quartiles. The upper whisker extends from the hinge to the largest value no further than 1.5 * IQR. The lower whisker extends from the hinge to the smallest value at most 1.5 * IQR.

d Expression score in Clone B (cluster C3 Log_2FC vs. C2) of HALLMARK Cell Cycle gene set (only differentially expressed 156 genes, two-sided $p \leq 0.01$). Lower heatmap shows expression levels in 9,534 cells from C1, C2, and C3 clusters.

Supplementary Fig. 7

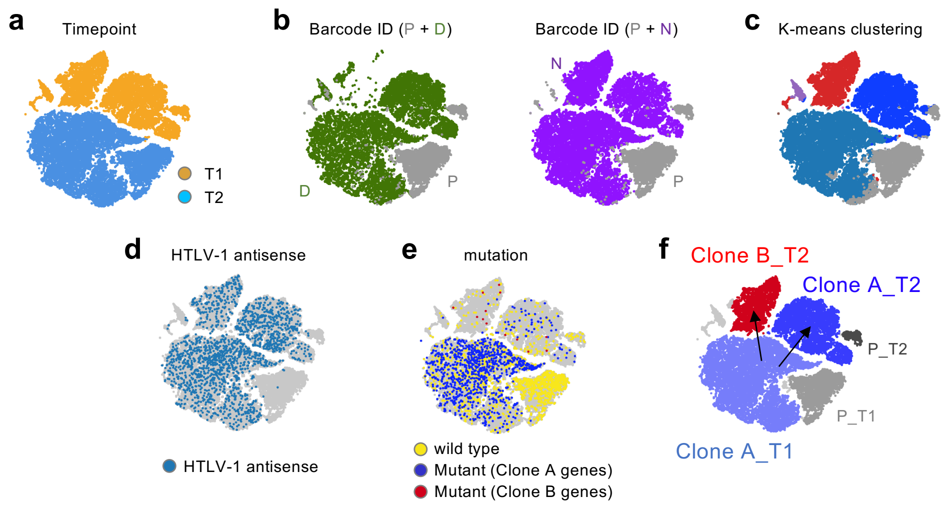

Supplementary Fig. 7 Longitudinal scRNA-seq analysis for ATL#6.
t-SNE projections of longitudinal scRNA-seq data (T1 and T2) in ATL#6 with cells colored according to timepoints (**a**), barcode ID (**b**), K-means clustering (**c**), viral antisense RNA (**d**), single-cell mutation detection (**e**), and assigned major clusters (**f**). The results of transcriptome analysis of these clusters are shown in Fig. 4k–m.

Supplementary Fig. 8

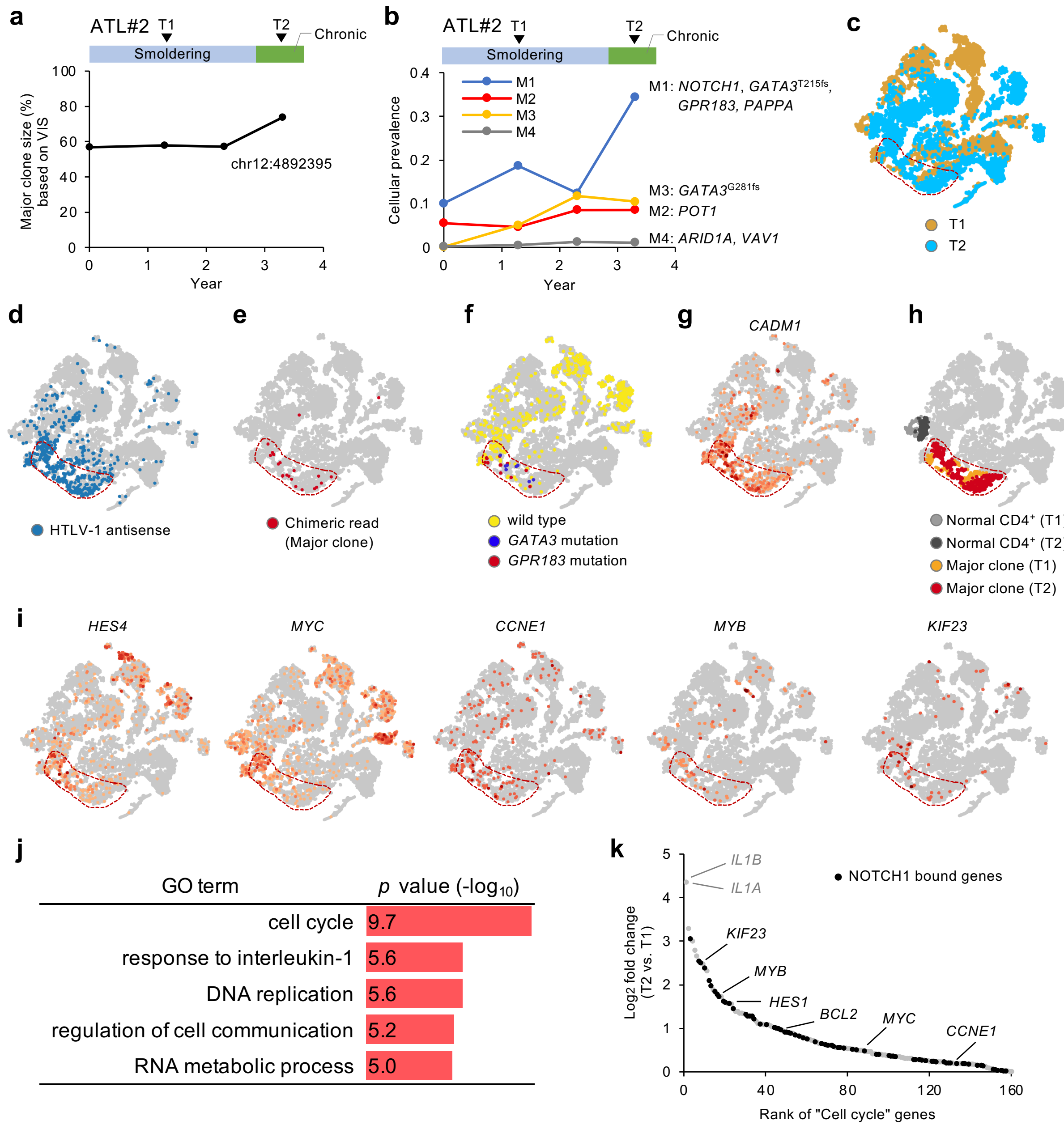

Supplementary Fig. 8 Longitudinal scRNA-seq analysis for ATL#2.
a Transition of clonality of a major clone calculated by VIS support tags.
b Line plot shows chronological transition of cellular prevalence calculated from the PyClone model.
c–i t-SNE projections of scRNA-seq data in ATL#2 with cells colored according to timepoints (**c**), viral antisense RNA (**d**), clone-specific chimeric transcripts (**e**), single-cell mutation detection (**f**), *CADM1* expression (**g**), assigned clusters of a major clone at T1 (orange) and T2 (red) ($p \leq 0.05$) (**h**), and expression of NOTCH1 target genes ($p \leq 0.05$) (**i**).
j Bar graph shows enriched gene ontologies with one-sided Fisher's exact p values ($-\text{Log}_{10}$) in major clone at T2 vs. T1.
k Scatter plot shows normalized Log_2 fold changes in major clone at T2 vs. T1. NOTCH1-bound genes defined using ChIP-seq data are colored in black.

Supplementary Fig. 9

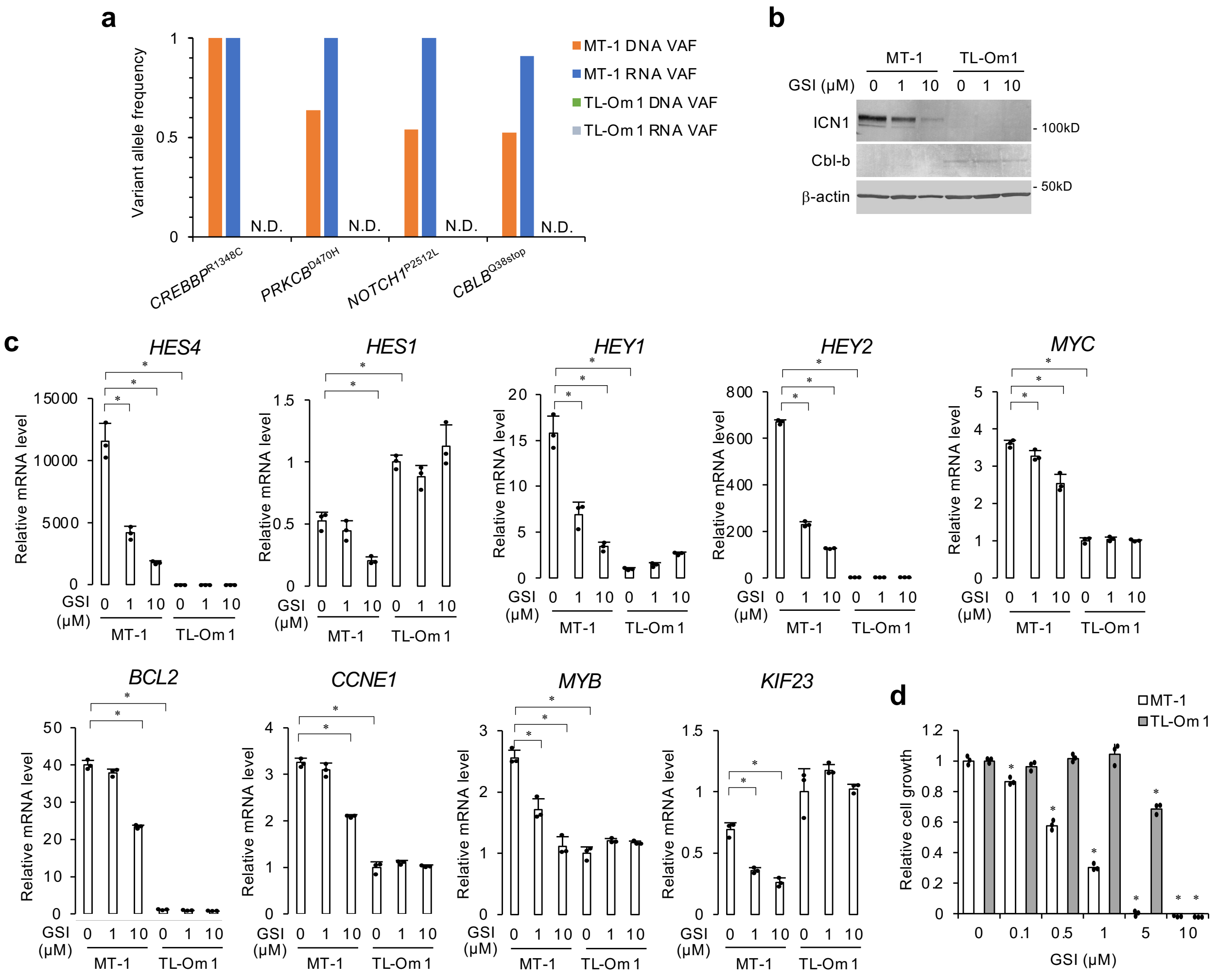

Supplementary Fig. 9 Biological function of mutant NOTCH1 in ATL cell line.

a Bar graph shows DNA and RNA VAF values of mutated genes found in MT-1 cells. N.D., not detected.

b Western blot panels show ICN1 level in MT-1 and TL-Om1 cells in the presence or absence of GSI for 24 h. Cbl-b was slightly detected only in TL-Om1. β-actin was used as an internal control. A representative result from three independent experiments is provided. Raw data are available from Source Data file.

c Bar graphs showed NOTCH1 target genes expression in cell lines with or without GSI for 24 h. n = 3 biologically independent samples, mean ± SD, $* \; p \leq 0.05$ (two-sided Student's *t*-test). Raw data are available from Source Data file.

d Bar graph shows result of cell proliferation assay with indicated does of GSI (μM) for 6 days. n = 3 biologically independent samples, mean ± SD, $* \; p \leq 0.05$ (two-sided Student's *t*-test). Raw data are available from Source Data file.

Supplementary Fig. 10

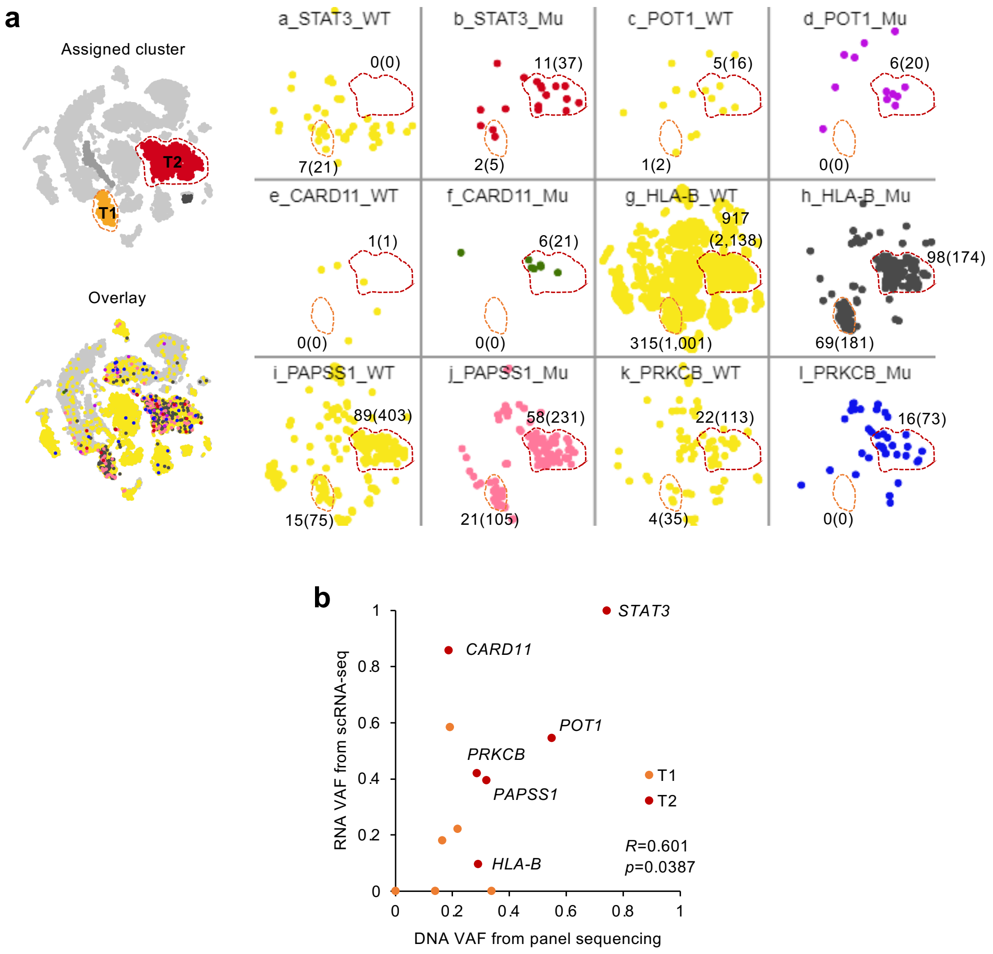

Supplementary Fig. 10 Longitudinal scRNA-seq analysis for ATL#3.

a t-SNE projections of scRNA-seq data in ATL#3, with cells colored according to single-cell genotype. Each cell was defined as a "mutant" (Mu, each color) if it contained at least one variant read, and a"wild-type" (WT, yellow) if only wild-type reads were detected. Genotyped cell numbers and supported tag numbers (in parentheses) detected in T1 and T2 clusters are annotated.

b Scatter plot shows relationship between DNA VAFs calculated in targeted DNA sequencing (x-axis) and RNA VAFs calculated in scRNA-seq (y-axis). A two-sided Spearman test was performed.

Supplementary Fig. 11

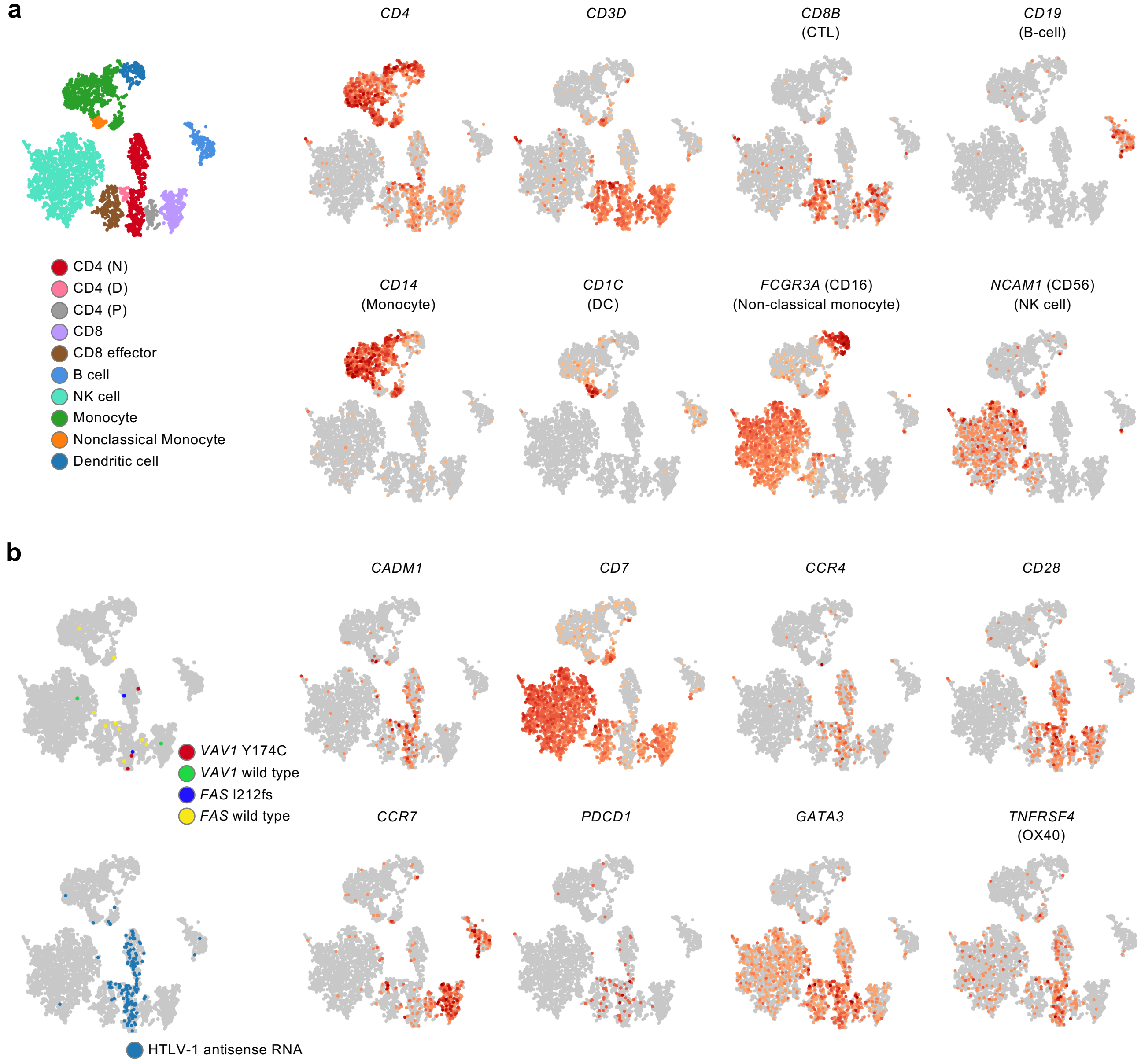

Supplementary Fig. 11 Cluster assignment in scRNA-seq for AC (AC#5).
a t-SNE projection of scRNA-seq data in AC#5, with cells colored according to lineage inference and expression of lineage-specific marker genes.
b t-SNE projection with cells colored according to single-cell mutation detection, viral antisense RNA, and key gene expression patterns in HTLV-1-infected cells.

Supplementary Fig. 12

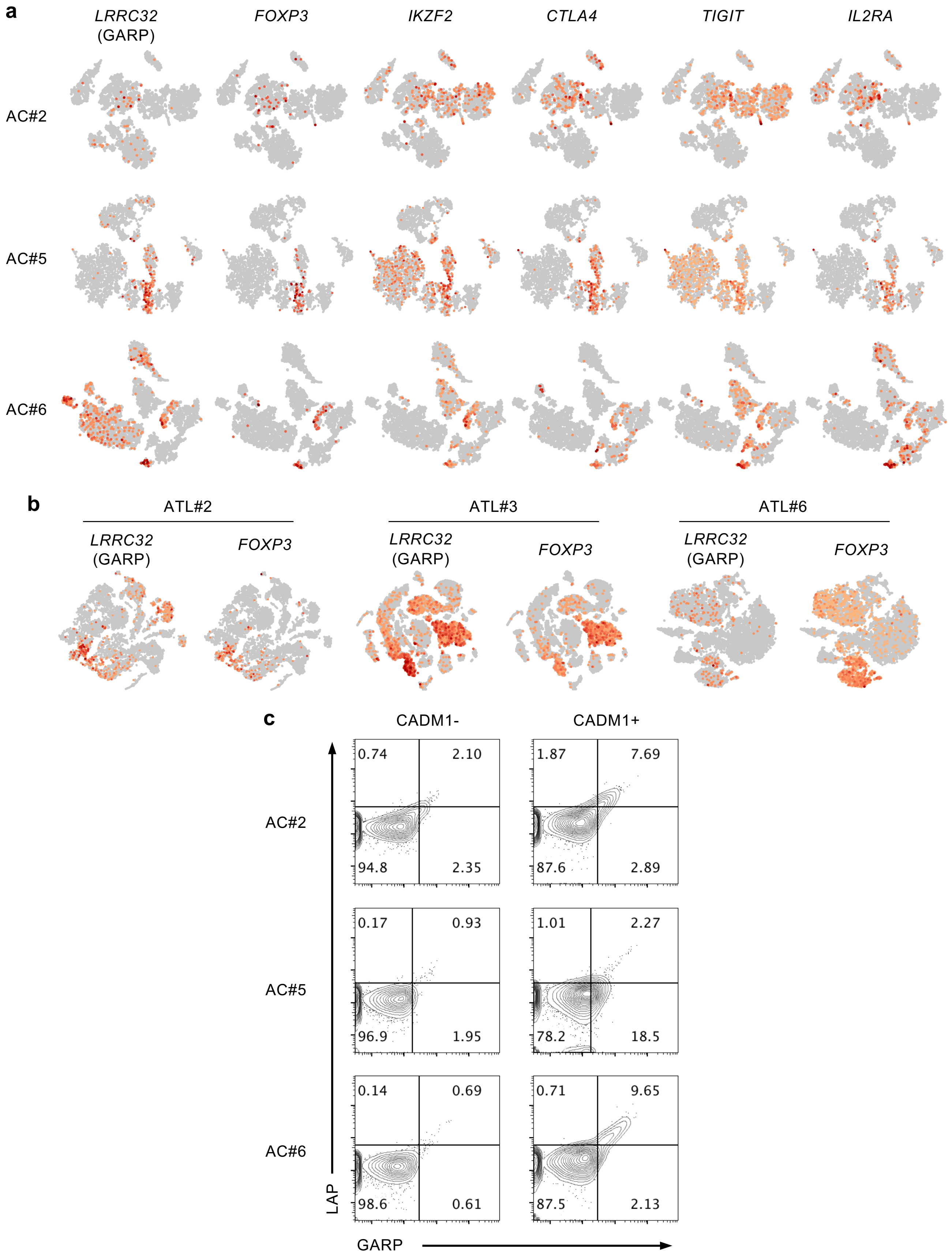

Supplementary Fig. 12 Expression of GARP and LAP in HTLV-1-infected cells in AC.
a t-SNE plots of AC#2, #5, and #6 show expression levels of Treg functional genes.
b t-SNE plots of ATL#2 (PBMC), ATL#3 (PBMC), and ATL#6 (P, D, N merged) show expression levels of *LRRC32* and *FOXP3*.
c GARP and LAP staining of PBMC from three ACs (AC#2, #5, #6) gated on $CD4^{+}/CADM1^{-}$ and $CD4^{+}/CADM1^{+}$ subpopulations. Quadrants are set based on background staining from isotype controls. Data summary is provided in Fig. 7h.

Supplementary Fig. 13

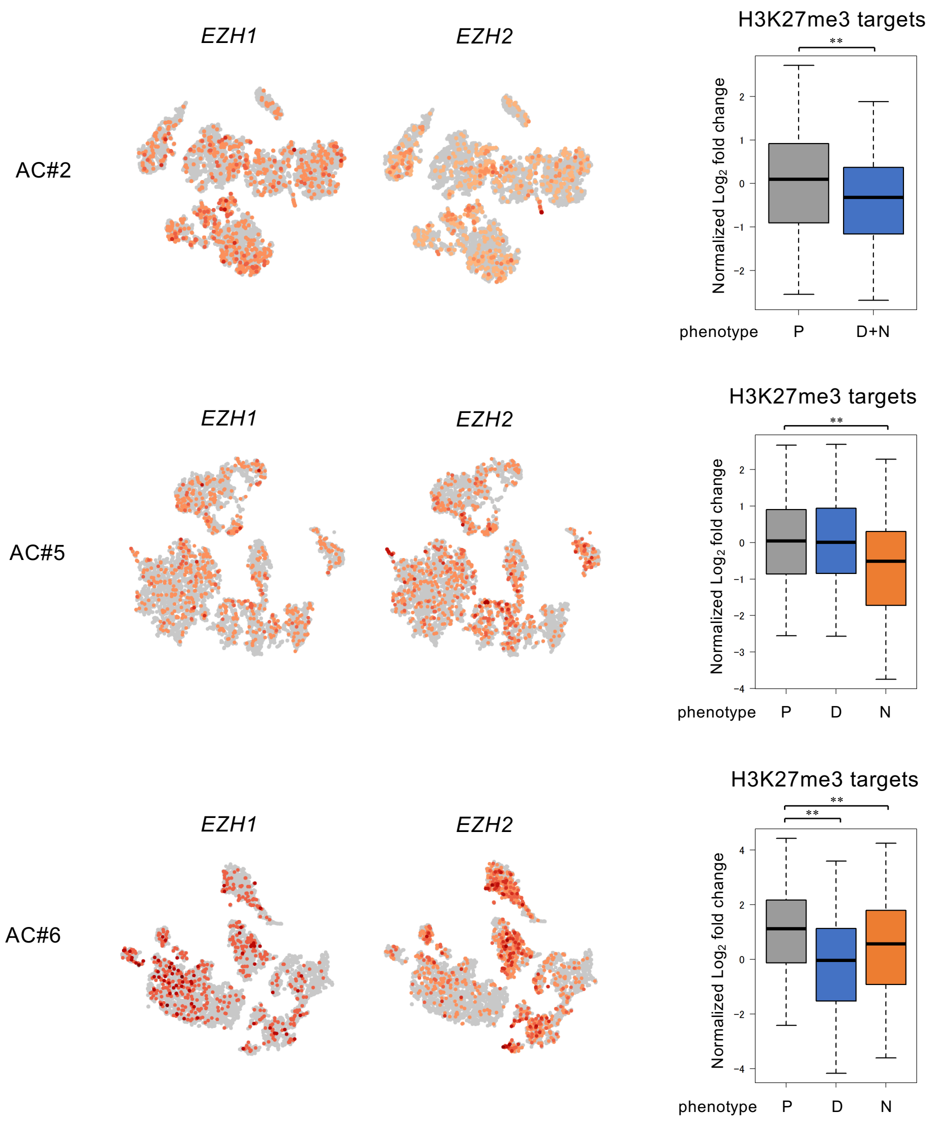

Supplementary Fig. 13 Silencing of H3K27me3 targets in HTLV-1-infected cells in AC.
Expression levels of *EZH1* and *EZH2* are overlaid on t-SNE plots in ACs (AC#2, #5, #6). Normalized Log_2 fold changes of H3K27me3 target genes (n = 609) in clustered P, D, and N subpopulations are shown in box plots. The middle lines within box plots correspond to the medians; lower and upper hinges correspond to first and third quartiles. The upper whisker extends from the hinge to the largest value no further than 1.5 * IQR. The lower whisker extends from the hinge to the smallest value at most 1.5 * IQR. ** $p \leq 1 \times 10^{-5}$ (two-sided Student's *t*-test).

Supplementary Table 1. Sequence status in WGS, WES, and HTLV-1/ATL panel

Cell line	Sequence platform	Infected cell	Unifected cell	Row fragment	Mapped fragment (hg38)	Duplicate rate	On target	Coverage (1x)	Human average depth	Provirus average depth
MT-2	WES	100%	0%	165,521,121	162,169,980	19%	81%	99.5%	297.05	-
TL-Om1	WES	100%	0%	207,608,037	203,230,408	20%	81%	99.4%	369.46	-
MT-2	WGS	100%	0%	311,058,915	297,795,491	7%	-	96.3%	16.00	22.00
TL-Om1	WGS	100%	0%	274,997,190	261,879,032	8%	-	96.1%	14.64	124.00
MT-2	Panel	1%	99%	42,849,937	38,868,854	74%	85%	99.7%	782.07	38.21
MT-2	Panel	5%	95%	39,647,071	37,667,448	43%	89%	99.8%	1700.49	433.87
MT-2	Panel	10%	90%	34,540,902	33,188,143	37%	90%	99.8%	1663.24	939.25
MT-2	Panel	20%	80%	37,156,073	35,353,491	33%	88%	99.8%	1841.26	2160.14
MT-2	Panel	50%	50%	52,645,821	50,522,388	43%	89%	99.8%	2234.63	4768.83
MT-2	Panel	100%	0%	36,107,431	33,995,195	28%	79%	99.7%	1680.04	10692.84
TL-Om1	Panel	1%	99%	33,376,378	31,365,176	45%	89%	99.8%	1374.17	10.92
TL-Om1	Panel	5%	95%	33,721,471	32,433,710	28%	87%	99.8%	1776.35	78.61
TL-Om1	Panel	10%	90%	40,899,562	39,588,860	30%	91%	99.8%	2161.42	179.68
TL-Om1	Panel	20%	80%	31,399,163	30,471,248	28%	89%	99.8%	1674.71	278.75
TL-Om1	Panel	50%	50%	37,089,678	35,986,550	34%	88%	99.8%	1800.86	1118.70
TL-Om1	Panel	100%	0%	35,399,073	34,152,970	38%	89%	99.8%	1620.11	2393.05

Supplementary Table 2. Viral integration sites in MT-2 and TL-Om1 cells

Cell Line	Locus	Support tag			Gene	
		WGS	WES	Panel		
MT-2	chr5:61535867-61535930	6	3	1763	Intronic	*ZSWIM6*
	chr6:134981852-134981908	11	1	1336	Intronic	*HBS1L*
	chr1:988700-989250	3	0	668	Intergenic	-
	chr11:118960220-118960242	5	3	1835	Intergenic	-
	chr19:48985620-48985698	10	42	1327	Intronic	*GYS1*
	chr2:147520320-147520354	10	1	1197	Intergenic	-
	chr15:24781435-24781512	6	0	1725	Intergenic	-
	chr4:167071306-167071372	3	0	176	Intronic	*SPOCK3*
	chr5:33657711-33657772	10	1	1649	Intronic	*ADAMTS12*
	chr9:85568210-85568284	5	0	873	Intronic	*AGTPBP1*
	chr15:43031617-43031666	3	0	932	Intronic	*UBR1*
	chr9:109399910-109399944	0	0	17	Intronic	*PTPN3*
	chr7:103304315-103304386	4	1	513	Intronic	*PMPCB*
	chr11:58061387-58061456	6	0	784	Intronic	*OR9Q1*
	chr8:128051546-128051618	0	0	16	Intronic	*PVT1*
	chr8:59059018-59059095	16	0	1750	Intronic	*TOX*
	chr19:48970662-48970711	3	3	618	Exon	*GYS1*
TL-Om1	chr1:121509466-121509525	7	0	1117	Intergenic	-

Supplementary Table 3. Clinical sample list and experimental platform.

Patient ID	Disease status	Timepoi	Platform_1 (Target-seq)	Platform_1 (Sample)	Platform_2 (bulk RNA-seq)	Platform_2 (Sample)	Platform_3 (scRNA-seq)	Platform_3 (Sample)	Figure	Supplementary Figure
ATL#1	Acute ATL	F1	Target-seq	PBMC					3	
	Acute ATL	F2	Target-seq	PBMC					3	
	Acute ATL	F3	Target-seq	PBMC					3	
	Acute ATL	F4	Target-seq	PBMC					3	
	Acute ATL	F5	Target-seq	PBMC					3	
	Acute ATL	F6	Target-seq	PBMC,P,N	bulk RNA-seq	N			3	1
ATL#2	Smoldering ATL	F1	Target-seq	PBMC						8
	Smoldering ATL	F2(T1)	Target-seq	PBMC,P,D,N	bulk RNA-seq	P,D,N	scRNA-seq	PBMC	3	1,8,12
	Smoldering ATL	F3	Target-seq	PBMC						8
	Chronic ATL	F4(T2)	Target-seq	PBMC			scRNA-seq	PBMC		8,12
ATL#3	Smoldering ATL	F1	Target-seq	PBMC					5	
	Smoldering ATL	F2	Target-seq	PBMC					5	
	Smoldering ATL	F3	Target-seq	PBMC					5	
	Smoldering ATL	F4	Target-seq	PBMC					5	
	Smoldering ATL	F5(T1)	Target-seq	PBMC,P,D,N	bulk RNA-seq	N	scRNA-seq	PBMC	3,5	1,10,12
	Chronic ATL	F6	Target-seq	PBMC					5	
	Acute ATL	F7(T2)	Target-seq	PBMC			scRNA-seq	PBMC	5	10,12
ATL#4	Smoldering ATL	-	Target-seq	P,D,N					3	
ATL#5	Chronic ATL	-	Target-seq	P,D,N					3	
ATL#6	Chronic ATL	T1	Target-seq	P,D,N			scRNA-seq	P,D,N	3	2,7,12
	Chronic ATL	T2	Target-seq	P,D,N	bulk RNA-seq	D,N	scRNA-seq	P,D,N	3	2,3,4,5,6,7,12
AC#1	Asymptomatic carrier	-	Target-seq	P,D,N					6	
AC#2	Asymptomatic carrier	-	Target-seq	P,D,N			scRNA-seq	PBMC	6,7	12,13
AC#3	Asymptomatic carrier	-	Target-seq	P,D,N					6	
AC#4	Asymptomatic carrier	-	Target-seq	P,D,N					6	
AC#5	Asymptomatic carrier	-	Target-seq	P,D,N			scRNA-seq	PBMC	6,7	11,12,13
AC#6	Asymptomatic carrier	-	Target-seq	P,D,N			scRNA-seq	PBMC	6,7	12,13
AC#7	Asymptomatic carrier	F1	Target-seq	PBMC					6	
	Acute ATL	F2	Target-seq	PBMC,P					6	
AC#8	Asymptomatic carrier	F1	Target-seq	PBMC					6	
	Smoldering ATL	F2	Target-seq	PBMC,P					6	
	Smoldering ATL	F3	Target-seq	PBMC					6	
	Smoldering ATL	F4	Target-seq	PBMC					6	
	Smoldering ATL	F5	Target-seq	PBMC					6	
	Smoldering ATL	F6	Target-seq	PBMC					6	
	Smoldering ATL	F7	Target-seq	PBMC					6	
	Smoldering ATL	F8	Target-seq	PBMC					6	
	Smoldering ATL	F9	Target-seq	PBMC					6	
	Smoldering ATL	F10	Target-seq	PBMC					6	
	Acute ATL	F11	Target-seq	PBMC					6	
AC#9	Asymptomatic carrier	F1	Target-seq	PBMC					6	
	Asymptomatic carrier	F2	Target-seq	PBMC					6	
	Asymptomatic carrier	F3	Target-seq	PBMC					6	
	Asymptomatic carrier	F4	Target-seq	PBMC					6	
	Smoldering ATL	F5	Target-seq	PBMC,P					6	
	Smoldering ATL	F6	Target-seq	PBMC					6	
	Acute ATL	F7	Target-seq	PBMC					6	
AC#10	Asymptomatic carrier	F1	Target-seq	PBMC					6	
	Asymptomatic carrier	F2	Target-seq	PBMC					6	
	Asymptomatic carrier	F3	Target-seq	PBMC					6	
	Asymptomatic carrier	F4	Target-seq	PBMC					6	
	Smoldering ATL	F5	Target-seq	PBMC,P					6	
	Chronic ATL	F6	Target-seq	PBMC					6	
	Chronic ATL	F7	Target-seq	PBMC					6	
	Acute ATL	F8	Target-seq	PBMC					6	

Supplementary Table 4. Primer list.

Quantitative PCR primer list (Target Gene: Sequence)

Gene symbol	Forward	Reverse
HES4	5′-tcgctcagctcaaaaccctca-3′	5′-gatgtccgccttctccagct-3′
HES1	5′-gaaatgacagtgaagcacctccg-3′	5′-cggtacttccccagcacact-3′
HEY1	5′-tcctgcagatgaccgtggatc-3′	5′-cccaaactccgatagtccatagca-3′
HEY2	5′-agcccgcccttgtcagtatc-3′	5′-ccccagggtcggtaaggtttatt-3′
BCL2	5′-tcgccctgtggatgactga-3′	5′-gggccgtacagttccacaaa-3′
MYC	5′-ccttgcagctgcttagacgc-3′	5′-tgaagctaacgttgaggggca-3′
CCNE1	5′-tcgctgatgaagatgcacac-3′	5′-gggagaggagaagccctattt-3′
MYB	5′-gcatcagaagatgaagacaatgttc-3′	5′-aggatgcaggttcccaggt-3′
KIF23	5′-atatcagcatcacgcacaacc-3′	5′-gctttcgagaaatcatccacac-3′
PRKCB	5′-ggagaaacttgaacgcaaagaga-3′	5′-tcgggaggtgttaggactgg-3′
BIRC5	5′-ggacagagaaagagccaagaaca-3′	5′-atggcacggcgcactttt-3′
CDC45	5′-gctgcacaaccattttgacct-3′	5′-gggaaataagtgcgtccagaaac-3′
AURKA	5′-ttagttgggaagcctccttttg-3′	5′-tctgttacaaagtcagggaatgtga-3′
AURKB	5′-agacctatcgccgcatcgt-3′	5′-ggttatgcctgagcagtttgg-3′
CDK1	5′-agcctagcatcccatgtcaa-3′	5′-cagaaattcgtttggctgga-3′
CCNB2	5′-acatggccaagaatgtggtg-3′	5′-atcttcaggagtttgctgcttg-3′
CDK4	5′-gtgcagtcggtggtacctga-3′	5′-gattcgcttgtgtgggttaaaag-3′
MKI67	5′-gcggagtgtcaagaggtgtg-3′	5′-cactgtccctatgacttctggttct-3′
TOP2A	5′-ttcgaaagcagtcacaagca-3′	5′-tacagattttgcccgaggag-3′
CDC20	5′-accagctagttatttggaagtaccc-3′	5′-catctgggctcatggtcag-3′
RPL19	5′-accaaggaagcacgcaagc-3′	5′-cagacaaagtgggaggttttatttc-3′

Primer sequences for point mutagenesis (Name: Sequence)

name	Forward	Reverse
VAV1 Y174C	5′-gagatctGtgaggacctcatgcgctc-3′	5′-gtcctcaCagatctcgtcgccttccg-3′
VAV1 M501R	5′-tttgagaGggccatctccaacatcta-3′	5′-gatggccCtctcaaactgctccatcc-3′

shRNA target sequences for lentiviral vector (Name: Sequence)

name	Sequence
shPRKCB#1	5′-gctgaaagaatcggacaaaga-3′
shPRKCB#2	5′-gacgacctgctttgattta-3′
