# Supplementary material for: Chronological genome and single-cell transcriptome integration characterizes the evolutionary process of adult T cell leukemia-lymphoma
Source: Nat Commun. 2021 Aug 10;12:4821. doi: 10.1038/s41467-021-25101-9 (PMC8355240; doi:10.1038/s41467-021-25101-9)
Supplement: Supplementary file 2 — Description of Additional Supplementary Files [file 41467_2021_25101_MOESM2_ESM.pdf]

Description of additional supplementary information

File name: Supplementary Data 1
Description: Gene list of HTLV-1/ATL panel.

File name: Supplementary Data 2
Description: Overall summary of scRNA-seq for ATL and AC.

File name: Supplementary Data 3
Description: Statistics of scRNA-seq data.

File name: Supplementary Data 4
Description: Summary of somatic mutations in AC cases.
