# Supplementary material for: Chronological genome and single-cell transcriptome integration characterizes the evolutionary process of adult T cell leukemia-lymphoma
Source: Nat Commun. 2021 Aug 10;12:4821. doi: 10.1038/s41467-021-25101-9 (PMC8355240; doi:10.1038/s41467-021-25101-9)
Supplement: Supplementary file 7 — Reporting Summary [file 41467_2021_25101_MOESM7_ESM.pdf]

nature research

Corresponding author(s): Kaoru Uchimaru, Yutaka Suzuki

Last updated by author(s): Jun 30, 2021

Reporting Summary

Nature Research wishes to improve the reproducibility of the work that we publish. This form provides structure for consistency and transparency in reporting. For further information on Nature Research policies, see our Editorial Policies and the Editorial Policy Checklist.

Statistics

For all statistical analyses, confirm that the following items are present in the figure legend, table legend, main text, or Methods section.

n/a	Confirmed	
☐	☒	The exact sample size (*n*) for each experimental group/condition, given as a discrete number and unit of measurement
☐	☒	A statement on whether measurements were taken from distinct samples or whether the same sample was measured repeatedly
☐	☒	The statistical test(s) used AND whether they are one- or two-sided<br>*Only common tests should be described solely by name; describe more complex techniques in the Methods section.*
☐	☒	A description of all covariates tested
☒	☐	A description of any assumptions or corrections, such as tests of normality and adjustment for multiple comparisons
☐	☒	A full description of the statistical parameters including central tendency (e.g. means) or other basic estimates (e.g. regression coefficient) AND variation (e.g. standard deviation) or associated estimates of uncertainty (e.g. confidence intervals)
☐	☒	For null hypothesis testing, the test statistic (e.g. *F*, *t*, *r*) with confidence intervals, effect sizes, degrees of freedom and *P* value noted<br>*Give P values as exact values whenever suitable.*
☐	☒	For Bayesian analysis, information on the choice of priors and Markov chain Monte Carlo settings
☒	☐	For hierarchical and complex designs, identification of the appropriate level for tests and full reporting of outcomes
☐	☒	Estimates of effect sizes (e.g. Cohen's *d*, Pearson's *r*), indicating how they were calculated

Our web collection on statistics for biologists contains articles on many of the points above.

Software and code

Policy information about availability of computer code

Data collection

Genomic analysis:
The sequence data were obtained using an HiSeq2500 sequencer (Illumina) with 100-bp paired-end reads. For whole-genome sequencing (WGS), TruSeq DNA Nano HT Library Prep Kit (Illumina) was used for library preparation following the manufacturer's instruction. The libraries were sequenced by the HiSeq3000 platform (Illumina) with 100-bp paired-end reads. For whole-exome sequencing (WES), SureSelelct XT HS Kit (Agilent Technologies) and SureSelect Human All Exon v5 (Agilent Technologies) were used for library preparation following the manufacturer's instruction. The libraries were sequenced by the HiSeq3000 platform (Illumina) with 100-bp paired-end reads.

RNA sequencing:
Total RNA of each sample was quantified and qualified by Agilent 2100 Bioanalyzer (Agilent Technologies). Sequencing was carried out using a 2×150bp paired-end configuration; image analysis and base calling were conducted by the HiSeq Control Software (HCS) + OLB + GAPipeline-1.6 (Illumina) on the HiSeq instrument.

Single-cell sequencing:
The single-cell RNA-seq library was constructed by using the Chromium Controller and Chromium Single Cell 5’ Reagent Kits and 3’ Reagent Kits v2 (10x Genomics) following the standard manufacturer’s protocols. To collect live cells for scRNA-seq, PBMC cryovials [1~10 × 106 cells / 1 mL of CELLBANKER 1 (ZENOAQ resource)] were removed from liquid nitrogen or −80 °C freezer and warmed in a 37 °C water bath. Cells were then pelleted by centrifugation at 500 × g for 5 min and resuspended in Phosphate-buffered saline (PBS). After twice washing with PBS, cells were then pipetted through a 40-μm filter to remove cell doublets and contamination. Cell viability (> 60%) was confirmed by trypan blue staining. The collected single-cell suspension from PBMCs or HAS-flow sorted PI-negative live cell subpopulations (1.6 × 104 live cells / sample) were immediately loaded onto the 10x Chromium controller in an effort to recover thousands of cells from each subpopulation for library preparation and sequencing.
Gel beads were prepared according to standard manufacturer’s protocols. Oil partitions of single-cell with oligo coated gel beads (GEMs) were captured and reverse transcription was performed, resulting in cDNA tagged with a cell barcode and unique molecular index (UMI). The library

was sequenced using the HiSeq3000 system (Illumina) according to the manufacturer's instruction. Sequencing was carried out using a 1 × 91~98bp single-end configuration, which is sufficient to align confidentially to the transcriptome (default setting).

Data analysis

Genomic analysis:
The sequenced data were aligned to the human reference genome hg38 by BWA (v0.7.15) software. The PCR duplicates were removed using Picard (v2.92) and SAMtools (v1.2) software. The somatic mutation candidates were called using MuTect2 from GATK (v4.0.12) software or VarScan2 and annotated with ANNOVAR (version 20191024). Candidate mutations with (i) ≥ 5 variant reads in tumor samples, (ii) a VAF in tumor samples ≥ 0.01, (iii) read depth ≥ 200, and (iv) tumor variant: normal variant ratio ≥ 2, were adopted and further filtered by excluding synonymous SNVs.

Clonality analysis:
The clonality analysis of HTLV-1-infected cells was performed by high-throughput sequencing based mapping of proviral integration sites. To designate the virus integration sites, sequence reads were aligned to human reference genome hg38 and virus genome (NC_001436.1) by BWA. Paired-end reads spanning the viral and human genomes and soft-clipped reads (> 15 bp soft-clipped region) were extracted using Perl scripts and then validated by Blastn (v2.6.0+). Clonality was calculated as population size of each clone by counting the extracted reads at host-provirus junction sites.

Subclonal analysis based on genomic data:
We used PyClone (v0.13.0) for analysis of subclonal population structure and reconstruct hierarchical trees. PyClone is based on a Bayesian clustering method, which uses a Markov chain Monte Carlo-based framework to estimate cellular prevalence values using somatic mutations. The somatic mutation candidates for PyClone were called using MuTect2, with (i) ≥ 5 variant reads in tumor samples, (ii) a VAF in tumor samples ≥ 0.05, (iii) read depth ≥ 200, and (iv) tumor variant: normal variant ratio ≥ 2. Clonal composition was investigated based on the beta binomial emission model, through which a set of clones with a discrete set of mutations (mutational clusters) were imputed together with their estimated clone size (cellular prevalence). Process of the clonal evolution were estimated by extrapolation of the estimated clone sizes at all tested timepoints. The hierarchical trees with imputed mutational subclusters were depicted by ClonEvol (v0.99.11) based on the results of clustering and cellular prevalence from the PyClone model. Integration site-based clonality was used as a parameter for clonal structure (polyclonal or monoclonal).

RNA sequencing:
For quality control, to remove technical sequences, including adapters, PCR primers, or fragments thereof, and quality of bases lower than 20, pass filter data of fastq format were processed by Trimmomatic (v0.30) to be high-quality clean data. For mapping, Hisat2 (v2.0.1) was used to index the reference genome sequence. Finally, clean data were aligned to the reference genome via software Hisat2. For differentially expressed gene analysis, HTSeq (v0.6.1) estimated gene and convert read counts to transcripts per million (TPM) from the pair-end clean data. Differentially expressed genes were selected based on the absolute log2 fold-change of ≥ 1. Selected genes were subjected to the hierarchical clustering analysis using iDEP.91 pipeline that contains DESeq2 package.

Single-cell RNA sequencing:
After sequencing analysis, fastq files were created by the Cell Ranger ver3.1.0 mkfastq pipeline (10x Genomics). The obtained fastq files were mapped to the reference genome provided by 10x Genomics (GRCh38). Cell Ranger count pipeline (v3.1.0) was used to perform demultiplexing, aligning reads, filtering, clustering, and gene expression analyses, using default parameters. Briefly, after read trimming, Cell Ranger used an aligner called STAR, which performs splicing-aware alignment of reads to the genome. Cell Ranger further aligned exonic and intronic confidently mapped reads to annotated transcripts by examining their compatibility with the transcriptome. Only uniquely mapping exonic reads were carried forward to UMI counting. After the UMI filtering steps with default parameters and expected cell counts, each observed barcode, UMI, gene combination was recorded as a UMI count in the feature-barcode matrix. The workflow also performed an improved Calling Cell Barcodes algorithm and identified the primary mode of high RNA content cells and also captured low RNA content cells. After data processing, we recovered quality-assured data for secondary analysis of gene expression.
To correct batch effects between timepoints, we used a Cell Range merge algorithm. To regress out the cell–cell variation in gene expression driven by batch and cluster data with corrected data in T1/T2 scRNA-seq data (ATL#3), we used standard Seurat v3 integration workflow with functions FindIntegrationAnchors() and IntegrateData(). The Cell Ranger data or batch-corrected data were imported into Loupe Cell Browser Software (v4.2.0) for t-distributed stochastic neighbor embedding (t-SNE) based clustering, heatmap generation, and gene expression distribution plots.

Single-cell mutation identification and analysis:
RNA variants from scRNA-seq data were validated from curated BAM files based on the results of Cell Ranger. For each cell barcode in the filtered Cell Ranger barcode list, and each somatic variant in the targeted sequencing data, variant bases were identified. Only reads that had both a Chromium Cellular Barcode (CB) tag and a Chromium Molecular Barcode (UB) tag were included. CB tags with the variant reads extracted by SAMtools were defined as at least one mutant read detected and mapped on each t-SNE projection using Loupe Cell Browser Software. Almost variants were validated by manual review to accurately identify mutant cells. One-sided Fisher exact tests were used to identify cell clusters that were enriched for somatic mutations.

Virus reads and host-virus chimeric reads from single-cell data:
For detection of virus reads, we processed Cell Ranger GRCh38-aligned sequence data. No-map and soft-clipped reads (> 20 bp soft-clipped) were extracted using Python scripts. The pass filter data of fastq format were processed to remove adopter and polyA sequences. The high-quality clean data were then aligned to human reference genome (hg38) and virus genome (NC_001436.1) via software STAR. For detection of cells expressing virus genes, CB tags with virus reads were defined as at least one virus read detected. Almost virus-aligned reads were derived from antisense strand. Both host- and virus-aligned soft-clipped reads were extracted as host-virus chimeric reads. Genomic breakpoints of chimeric reads were analyzed from supplementarily mapped data from STAR alignment to link the clone-specific chimeric reads with the VISs identified in the corresponding clones. The extracted CB tags with virus antisense reads or clone-specific host-virus chimeric reads were mapped on t-SNE projection using Loupe Cell Browser. One-sided Fisher exact tests were used to identify cell clusters that were enriched for virus reads.

Cluster assignment and single-cell expression analysis:

Expression patterns of CD4, CADM1, and CD7 were used and overlaid on the t-SNE to identify HTLV-1-infected subpopulations. CBs with HTLV-1-derived antisense transcripts were also overlaid on the t-SNE. Stable expression of HTLV-1 antisense RNA (predominantly HBZ) served for inference of infected cells ($p \leq 0.05$). Infected clone-specific host-virus chimeric reads were significantly enriched in each cluster ($p \leq 0.05$). To detect the mutation-harboring clones estimated by PyClone, RNA variants from scRNA-seq data were validated from curated BAM files based on the results of Cell Ranger. CB tags with variant reads were defined as at least one mutant read detected and mapped on each t-SNE projection.
For analysis of scRNA-seq from PBMCs, major cell types were annotated by marker genes, including CD4+ T-cell (CD3D+, CD4+), CD8+ T-cell (CD3D+, CD8B+), CD8+ effector CTL (CD3D+, CD8B+, KLRB1+, CCR7−), NK cell [NCAM1+ (CD56)], B cell (CD79A+, CD19+), monocyte (CD14+), nonclassical monocyte [FCGR3A+ (CD16)], and dendritic cell (DC) (CD1C+). The assigned lineage clusters were mapped on t-SNE projection using Loupe Cell Browser.
Log2 fold-change and median-normalized average values of assigned clusters were obtained via Loupe Cell Browser and used in following analysis of differentially expressed genes within each cluster. Manual clustering based on expression patterns was curated by original Python scripts or polygonal selection tool (Loupe Cell Browser interface).

Bioinformatic analysis and statistics:
ntegrative Genomics Viewer (IGV) tool was used for visualizing and interpreting the results of DNA-seq and RNA-seq. For differentially expressed gene analysis, HTSeq (v0.6.1) estimated gene and convert read counts to transcripts per million (TPM) from the pair-end clean data. Differentially expressed genes were selected based on the absolute log2 fold-change of ≥ 1. Selected genes were subjected to the hierarchical clustering analysis using iDEP.91 pipeline that contains DESeq2 package. Gene set enrichment analysis (GSEA) was performed using GSEA software (v4.0.3) (http://www.broadinstitute.org/gsea) with 1,000 permutations. Gene sets used in this study were selected from the MSigDB hallmark gene sets (http://www.broadinstitute.org/gsea/msigdb/collections.jsp). Significantly enriched gene sets were evaluated by normalized enrichment score (NES) and nominal p value ($p < 0.001$). Gene Ontology analysis was performed by DAVID Bioinformatics Resources (https://david.ncifcrf.gov/). For target gene analysis of NOTCH1, STAT3, RBPJ, and NFAT, enrichment data and bigwig format from previous ChIP-seq studies were obtained from the ChIP-Atlas database (https://chip-atlas.org/)58. Gene lists of NOTCH1 and STAT3 targets were created by integrating of hallmark genes and ChIP bound genes. Significant differences in gene expression and other biological assays between the two groups were analyzed by Student's t-test. Correlations between two groups were analyzed by Pearson's correlation coefficients and probabilities of overlap between gene sets were statistically tested. Box plots, hierarchical clustering, and correlation matrix were analyzed and visualized by using R (v3.2.3). The flow-cytometry data were analyzed by FlowJo software (v9.6, v10.6.1) (Tree Star).

For manuscripts utilizing custom algorithms or software that are central to the research but not yet described in published literature, software must be made available to editors and reviewers. We strongly encourage code deposition in a community repository (e.g. GitHub). See the Nature Research guidelines for submitting code & software for further information.

Data

Policy information about availability of data

All manuscripts must include a data availability statement. This statement should provide the following information, where applicable:

- Accession codes, unique identifiers, or web links for publicly available datasets
- A list of figures that have associated raw data
- A description of any restrictions on data availability

All sequencing data, including Target-seq, RNA-seq, and scRNA-seq, have been deposited in the National Bioscience Database Center (NBDC) Human Database (https://humandbs.biosciencedbc.jp/.), which is associated with DNA DataBank of Japan (DDBJ) under an accession number JGAS000301 (https://ddbj.nig.ac.jp/resource/jga-study/JGAS000301). Raw experimental data from western blots, luciferase assay, Venus competition assay, cell growth assay, and qRT-PCR are available from Source Data file. Other intermediate files from the sequencing data are available from the corresponding authors. Gene expression dataset of the TCR–NFAT pathway targets (GSE13738) were obtained from Gene Expression Omnibus (GEO) (https://www.ncbi.nlm.nih.gov/geo/query/acc.cgi?acc=GSE13738). For target gene analysis of transcription factors, enrichment data and bigwig format from previous ChIP-seq studies were obtained from the ChIP-Atlas database (https://chip-atlas.org/). NOTCH1, SRX070882; RBPJ, SRX070884; STAT3, SRX5801455; NFAT, SRX3279752.

Field-specific reporting

Please select the one below that is the best fit for your research. If you are not sure, read the appropriate sections before making your selection.

☒ Life sciences ☐ Behavioural & social sciences ☐ Ecological, evolutionary & environmental sciences

For a reference copy of the document with all sections, see nature.com/documents/nr-reporting-summary-flat.pdf

Life sciences study design

All studies must disclose on these points even when the disclosure is negative.

Sample size	No statistical methods were used to determine sample size since this is an exploratory study. Sample size was thus determined by the availability of patient recruitment. We enrolled individuals who provided consent for our study during the enrollment period between 1/Sep/2007 and 31/Mar/2020. List of used clinical samples are provided in Supplementary Table. The sample size of our study was sufficient to assess our sequencing data and to confirm novel findings with statistical significance.
Data exclusions	All data is included in the current study.
Replication	The experimental findings were reliably and independently reproduced. The replication numbers were described in the corresponding figure

legends.

Randomization

Not applicable since this is a case-series study which was therefore not planned to detect any difference in effects between the cohorts with and without intervention. Samples of other experiments were also not randomized into experimental groups. Randomization was not relevant to the study design.

Blinding

Blinding was not relevant to our study with clinical samples because it was essential to understand underlying confounding variables in our associations, such as clinical subtype, sex, etc. For other experiments, we were not blinded to group allocation.

Reporting for specific materials, systems and methods

We require information from authors about some types of materials, experimental systems and methods used in many studies. Here, indicate whether each material, system or method listed is relevant to your study. If you are not sure if a list item applies to your research, read the appropriate section before selecting a response.

Materials & experimental systems

n/a	Involved in the study	
☐	☒	Antibodies
☐	☒	Eukaryotic cell lines
☒	☐	Palaeontology and archaeology
☒	☐	Animals and other organisms
☐	☒	Human research participants
☒	☐	Clinical data
☒	☐	Dual use research of concern

Methods

n/a	Involved in the study	
☒	☐	ChIP-seq
☐	☒	Flow cytometry
☒	☐	MRI-based neuroimaging

Antibodies

Antibodies used

For flow-cytometory, an unlabeled CADM1 antibody (CM004-6, clone 3E1) and an isotype control chicken immunoglobulin Y (IgY) antibody (2:100) were purchased from MBL. These were biotinylated (primary amine biotinylation) using biotin N-hydroxysuccinimide ester (Sigma-Aldrich). Pacific Orange-conjugated anti-CD14 antibody (MHCD1430, clone TuK4) was purchased from Invitrogen. All other antibodies were obtained from BioLegend. Cells were stained using a combination of biotin-CADM1 (1:100), allophycocyanin (APC)-CD7 (clone CD7-6B7, 5:100), APC-Cy7-CD3 (clone SK7, 5:100), Pacific Blue-CD4 (clone RPA-T4, 5:100), and Pacific Orange-CD14. APC-GARP (clone 7B11, 5:100) and FITC-LAP (clone TW7-16B4, 5:100) antibodies were used instead of CD7 antibody for GARP/LAP co-staining in CD4+/CADM1− and CD4+/CADM1+ subpopulations. After washing, phycoerythrin (PE)-conjugated streptavidin (SA10041, , 2:100, Thermo Fisher Scientific) was applied.

For western blotting, protein levels of ICN1 and Cbl-b were analyzed with primary antibodies, as follows; ICN1 (Cleaved Notch1 (V1754), #4147, 1:1000, Cell Signaling Technology), Cbl-b (sc-8006, 1:200, Santa Cruz), and b-actin (sc-69879, 1:1000, Santa Cruz). VAV1 expression was validated by immunoblotting with antibodies against VAV1 (#4657, 1:1000, Cell Signaling Technology) and HA (code 561, 1:1000, MBL). Alkaline phosphatase-conjugated anti-mouse (S3721, Promega) and anti-rabbit (S3731, Promega) secondary antibodies and BCIP/NBT substrate (S3771, Promega) were used for detection.

For TCR stimutation, the cells were seeded in 48-well plates and stimulated with or without 1 ug/mL plate-immobilized anti-CD3 monoclonal antibody (130-093-387, Miltenyi Biotec) for 20 hours.

Validation

All antibodies used were validated for their use in flow-cytometory and western blotting experiments with human samples, as shown on the website provided by the respective companies.

Eukaryotic cell lines

Policy information about cell lines

Cell line source(s)

The HTLV-1-infected cell line MT-2 and ATL-derived cell line MT-1 were kindly provided from an established researcher Dr. Miyoshi. ATL-derived TL-Om1 cells were kindly provided from an established researcher Dr. Sugamura. ATL-derived KOB cells were kindly provided from an established researcher Dr. Kamihira. Jurkat cells were purchased from RIKEN BRC cell bank (RCB3052).

Authentication

The cell lines were verified by RIKEN BRC or established researchers and monitored for cross-contamination. This study authenticated the provirus integration sites and somatic mutations of the HTLV-1-infected cell lines by panel-based targeted sequencing. Cell surface expressions of CD4 and CADM1 were validated by flow-cytometory.

Mycoplasma contamination

Cells were tested for mycoplasma contamination using mycoplasma detection PCR and were found to be negative for

	mycoplasma contamination.
Commonly misidentified lines (See ICLAC register)	Commonly misidentified lines have not been used in this study.

Human research participants

Policy information about studies involving human research participants

Population characteristics	All patients with ATL were categorized into clinical subtypes, according to Shimoyama's criteria. Patients with various complications, such as autoimmune disorders and systemic infections, were excluded. Lymphoma-type patients were also excluded because ATL cells are not considered to exist in the peripheral blood of this clinical subtype. Written informed consent was obtained from all patients and asymptomatic individuals. Because of small sample-size, this study did not consider the covariate-relevant population characteristics of the human research participants.
Recruitment	Peripheral blood samples were collected from in-patients and out-patients at IMSUT Hospital, The Institute of Medical Science, The University of Tokyo. No statistical methods were used to determine sample size since this is an exploratory study. We enrolled individuals who provided consent for our study during the enrollment period between 1/Sep/2007 and 31/Mar/2020. We did not use other criteria for the recruitment. A part of genomic DNA from ATL and asymptomatic HTLV-1 carriers were also collected with informed consent as a collaborative project of the Joint Study on Predisposing Factors of ATL Development (JSPFAD).
Ethics oversight	The present study was approved by the Institutional Review Board of our institute (the University of Tokyo, Tokyo, Japan).

Note that full information on the approval of the study protocol must also be provided in the manuscript.

Flow Cytometry

Plots

Confirm that:

- [x] The axis labels state the marker and fluorochrome used (e.g. CD4-FITC).
- [x] The axis scales are clearly visible. Include numbers along axes only for bottom left plot of group (a 'group' is an analysis of identical markers).
- [x] All plots are contour plots with outliers or pseudocolor plots.
- [x] A numerical value for number of cells or percentage (with statistics) is provided.

Methodology

Sample preparation	Single-cell suspensions of lymphocytes were stained with fluorescent-labeled antibodies. Cells were stained using a combination of biotin-CADM1, allophycocyanin (APC)-CD7, APC-Cy7-CD3, Pacific Blue-CD4, and Pacific Orange-CD14. APC-GARP and FITC-LAP antibodies were used instead of CD7 antibody for GARP/LAP co-staining in CD4+/CADM1− and CD4+/CADM1+ subpopulations. After washing, phycoerythrin (PE)-conjugated streptavidin was applied. Propidium iodide was added to the samples to stain dead cells immediately before flow cytometry.<br>PBMCs from ATL patients and HTLV-1 carriers including longitudinal samples were sorted into HTLV-1-infected cell population (CD4+/CADM1+/CD7+), a more aggressive cell population (CD4+/CADM1+/CD7−), and HTLV-1-uninfected T-cells (CD4+/CADM1−/CD7+).
Instrument	A FACSAria II instrument (BD Biosciences) was used for all multicolor flow cytometry and fluorescence-activated cell sorting.
Software	The collected data were analyzed by FlowJo software (v9.6, v10.6.1) (Tree Star).
Cell population abundance	After cell sorting, we were able to assess purity in our cell sorts by assessing HTLV-1 provirus PCR. In addition, expression pattern of cell surface markers such as CADM1 and CD7 were validated using RNA-seq data.
Gating strategy	Gating was determined using fluorescent-minus-one controls for each color used in each FACS experiment to ensure that positive populations were solely associated with the antibody for that specific marker.<br>PBMCs from ATL patients and HTLV-1 carriers including longitudinal samples were sorted into HTLV-1-infected cell population (CD4+/CADM1+/CD7+), a more aggressive cell population (CD4+/CADM1+/CD7−), and HTLV-1-uninfected T-cells (CD4+/CADM1−/CD7+).

- [x] Tick this box to confirm that a figure exemplifying the gating strategy is provided in the Supplementary Information.
